# Hippocampal Theta Input to the Amygdala Shapes Feedforward Inhibition to Gate Heterosynaptic Plasticity

**DOI:** 10.1016/j.neuron.2015.08.024

**Published:** 2015-09-23

**Authors:** Michaël Bazelot, Marco Bocchio, Yu Kasugai, David Fischer, Paul D. Dodson, Francesco Ferraguti, Marco Capogna

**Affiliations:** 1 MRC Brain Network Dynamics Unit, Department of Pharmacology, University of Oxford, Mansfield Road, Oxford OX1 3TH, UK; 2 Department of Pharmacology, Innsbruck Medical University, Peter Mayr Straße 1a, 6020 Innsbruck, Austria

## Abstract

The dynamic interactions between hippocampus and amygdala are critical for emotional memory. Theta synchrony between these structures occurs during fear memory retrieval and may facilitate synaptic plasticity, but the cellular mechanisms are unknown. We report that interneurons of the mouse basal amygdala are activated during theta network activity or optogenetic stimulation of ventral CA1 pyramidal cell axons, whereas principal neurons are inhibited. Interneurons provide feedforward inhibition that transiently hyperpolarizes principal neurons. However, synaptic inhibition attenuates during theta frequency stimulation of ventral CA1 fibers, and this broadens excitatory postsynaptic potentials. These effects are mediated by GABA_B_ receptors and change in the Cl^−^ driving force. Pairing theta frequency stimulation of ventral CA1 fibers with coincident stimuli of the lateral amygdala induces long-term potentiation of lateral-basal amygdala excitatory synapses. Hence, feedforward inhibition, known to enforce temporal fidelity of excitatory inputs, dominates hippocampus-amygdala interactions to gate heterosynaptic plasticity.

**Video Abstract:**

## Highlights

• Theta stimulation of CA1 ventral hippocampal fibers activates amygdala interneurons
• Interneurons induce feedforward inhibition that hyperpolarizes principal neurons
• Theta-evoked inhibition attenuates to broaden excitation on principal neurons
• Feedforward inhibition gates heterosynaptic plasticity via GABA_B_ receptors

## Teaser

Hippocampal-amygdala interactions are critical for emotional memory, but the cellular mechanisms are unknown. In this paper, Bazelot, Bocchio et al. functionally demonstrate that GABAergic neurons of the basal amygdala gate principal neuron firing and heterosynaptic plasticity in the mouse amygdala.

## Introduction

The ventral hippocampus (vHPC) and the basolateral amygdala (BLA) are part of an extensive neural circuit encoding emotional memories (Fanselow, 2010; Maren and Quirk, 2004). This circuit can be dysfunctional in neuropsychiatric disorders in humans (Phelps, 2004; Richardson et al., 2004) and in animal models (Ghosh et al., 2013; Santos et al., 2013; Zhang et al., 2014). Optogenetic inhibition of hippocampal CA1 pyramidal cells impairs contextual fear memory acquisition and retrieval (Goshen et al., 2011). The disconnection of the vHPC to the BLA prevents the renewal of an extinguished fear memory (Orsini et al., 2011). This suggests that the vHPC gates fear behavior by sending contextual information to the BLA (Maren et al., 2013). Additionally, optogenetic manipulation of the projections from the BLA to the vHPC shows that these two structures are involved in anxiety-related behavior (Felix-Ortiz et al., 2013) and social behavior (Felix-Ortiz and Tye, 2014).

Synchronous oscillatory activity between HPC and BLA represents an intermediate phenomenon to link the firing of single neurons to behavior (Paré et al., 2002). Theta synchronization of lateral amygdala (LA) and CA1 HPC increases during fear memory retrieval in rodents (Seidenbecher et al., 2003), and the degree of theta synchrony predicts memory performance after fear conditioning (Popa et al., 2010). Likewise, dynamic shifts in theta synchrony of CA1, BLA, and medial prefrontal cortex (mPFC) are associated with ongoing defensive behavior (Likhtik et al., 2014) and extinction of conditioned fear (Lesting et al., 2011).

What cellular mechanisms orchestrate vHPC-BLA interactions? Neurons of the BLA fire phase-locked to entorhinal (Paré and Gaudreau, 1996) and hippocampal theta oscillations (Bienvenu et al., 2012), suggesting that the firing of single neurons of the BLA could be synchronized by external theta inputs. However, the mechanisms through which CA1 inputs recruit BLA neurons to drive network synchronization remain unknown. Anatomical work demonstrated reciprocal connections between the ventral CA1 (vCA1) and the BLA (Pitkänen et al., 2000). In particular, the basal (BA) and basomedial (BM) nuclei of the amygdala are the most densely innervated by the vCA1 (Kishi et al., 2006; Müller et al., 2012). At the single cell level, individual vCA1 pyramidal neurons project to several areas, including the BLA and mPFC (Arszovszki et al., 2014; Ciocchi et al., 2015; Ishikawa and Nakamura, 2006), and these neurons are preferentially activated by fear renewal (Jin and Maren, 2015). It is thought that vCA1 pyramidal neurons modulate the activity of BLA principal neurons (PNs), as “fear neurons” have been shown to receive functional input from this area (Herry et al., 2008). We previously reported that anatomically identified BLA interneuron (IN) populations preferentially fire in phase with hippocampal theta oscillations in vivo (Bienvenu et al., 2012; Mańko et al., 2012). These observations prompted us to hypothesize that GABAergic INs of the BA would gate excitatory input from vCA1 to control the firing and synaptic plasticity of PNs. To test this hypothesis, we combined in vivo and ex vivo recordings from single neurons of the BA and optical stimulation of vCA1 pyramidal cell axons. We found that feedforward inhibition (FFI) dynamics gate vHPC input and heterosynaptic plasticity via GABA_B_-receptor-dependent mechanisms and change in Cl^−^ driving force.

## Results

### Differential Firing of PNs and INs of the BA during Theta Oscillations In Vivo

**Figure 1:**
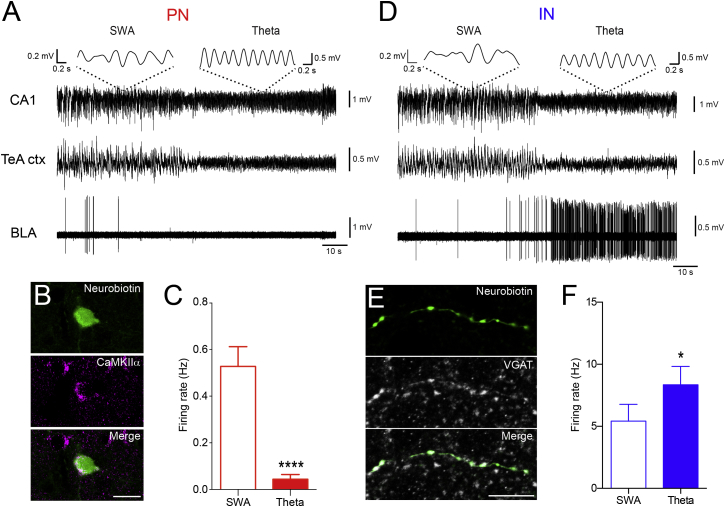
Differential Firing Frequencies of PNs and INs during Theta Epochs (A) In vivo single unit recording from a representative PN of the BA during SWA and theta oscillations recorded from CA1 HPC and TeA cortex (ctx). This cell became silent at the onset of theta oscillations. Insets show 2 s of CA1 LFP trace band-pass filtered between 2 and 3 Hz (SWA, left) and 3 and 6 Hz (theta oscillations, right). (B) Juxtacellularly labeled PN displaying immunoreactivity for CaMKIIα. (C) PNs fired at higher frequencies during cortical and hippocampal SWA (0.52 ± 0.08 Hz) and at significantly lower frequencies during theta oscillations (0.04 ± 0.02 Hz, ^∗∗∗∗^p < 0.0001 n = 16). (D) In vivo single unit recording from an IN of the BA during SWA and theta oscillations recorded from CA1 HPC and TeA. The firing frequency increased during theta oscillations. Insets: 2 s of CA1 LFP trace band-pass filtered between 2 and 3 Hz (SWA, left) and 3 and 6 Hz (theta oscillations, right). (E) Juxtacellularly labeled GABAergic IN displaying axonal immunoreactivity for VGAT. (F) INs fired at lower frequencies during cortical and hippocampal SWA (5.42 ± 1.35 Hz) and at significantly higher frequencies during theta oscillations (8.35 ± 1.48 Hz, ^∗^p < 0.05, n = 20). Scale bars: (B), 20 μm; (E), 10 μm. Data are presented as means ± SEM.

First, we set out to investigate the activity of PNs and GABAergic INs of the BA when theta oscillations occur in areas interconnected with the amygdala. We recorded the firing of single neurons of the BA and the local field potential (LFP) in CA1 and/or Temporal associative cortex (TeA), two structures projecting to the BLA, in urethane-anesthetized mice. In the BA, PNs (n = 27) and INs (n = 25) could be separated on the basis of their spike waveforms and firing regularity (Figures S1A–S1C; Experimental Procedures), consistent with previous observations (Bienvenu et al., 2012). Immunoreactivity for CaMKIIα (14/14) and VGAT (10/10) was confirmed for a subset of PNs and INs juxtacellularly labeled after their recording (Figures 1B and 1E, respectively). The firing rates of the recorded neurons were cell type specific and brain state dependent, consistent with previous data obtained in awake cats (Paré and Gaudreau, 1996). Specifically, BA PNs fired at higher frequencies during cortical and hippocampal slow wave activity (SWA) (0.52 ± 0.08 Hz) and at significantly lower frequencies during theta oscillations (0.04 ± 0.02 Hz, p < 0.0001, n = 16; Figures 1A and 1C). In contrast, INs fired at lower rates during cortical and hippocampal SWA (5.42 ± 1.35 Hz) and at significantly higher rates during theta oscillations (8.35 ± 1.48 Hz, p < 0.05, n = 20; Figures 1D and 1F). Although most of the INs (16/20) increased their firing rates during theta episodes, a few (4/20) showed a reduction. These results suggest that the firing of BA PNs might be more tightly controlled by GABAergic INs during theta epochs compared to slow wave states.

### Theta Burst Stimulation of vCA1 Pyramidal Cell Axons Inhibits BA PNs and Activates BA INs In Vivo

**Figure 2:**
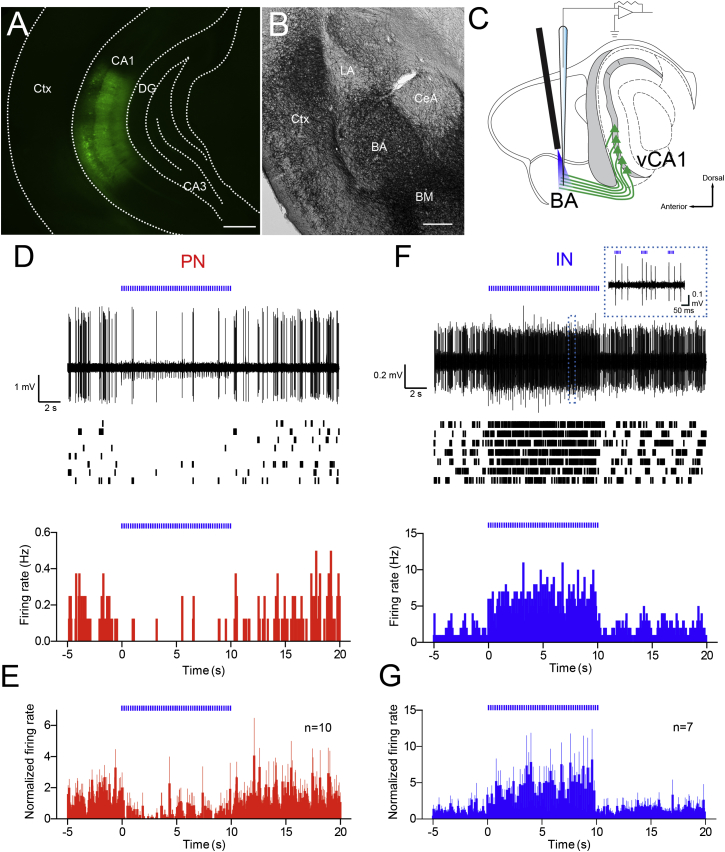
Optical TBS of Hippocampal Axons Inhibits PNs and Triggers Firing in INs of the BA In Vivo (A) Expression of ChR2/YFP in ventral/intermediate CA1. (B) Light micrograph showing ChR2/YFP-positive axons in the amygdaloid complex. BA and BM nuclei showed densest innervation, whereas only few fibers were detectable in the LA and CeA. (C) Scheme showing experimental configuration: single unit recordings and juxtacellular labeling from neurons of the BA and photostimulation of vCA1 pyramidal cells axons through a fiber optic implanted above the BA. (D) Representative PN was inhibited by optical TBS. Superimposed traces (n = 8, top), singularly represented in rasterplot (middle), and peri-stimulus time histogram (PSTH) (50-ms bins, bottom). Note spikelet-like events during TBS are stimulation artifacts. (E) Normalized firing rates (50-ms bins) of BA PNs in response to optical TBS of hippocampal axons (baseline versus TBS, p < 0.01, n = 10). (F) Representative IN was activated by optical TBS. Superimposed traces (n = 8, top), singularly represented in raster plot (middle) and PSTH (50-ms bins, bottom). Inset: TBS evokes action potentials primarily following the first pulse of each train. (G) Normalized firing rates (50 ms bins) of BA INs in response to optical TBS of hippocampal axons (baseline versus TBS, p < 0.05, n = 7). Scale bars: (A), 350 μm; (B), 250 μm. Data are presented as means ± SEM.

We then sought to understand the influence of a theta frequency input from vCA1 pyramidal cells on the firing of BA neurons. To this aim, we transfected pyramidal cells of vCA1 with the ultrafast (E123T/T159C) Channelrhodopsin-2 (ChR2) (Figures 2A and S2), which more efficiently evokes action potentials at high frequency stimulation compared to the common H134R variant (Berndt et al., 2011). Three to four weeks after viral injection, ChR2+ axons densely innervated the BA and BM, together with the external and intermediate capsules surrounding the BLA, whereas only sparse fibers were observed in the LA (Figure 2B). This pattern of innervation was consistent with previous tracing studies in rodents (Kishi et al., 2006; Müller et al., 2012).

To optically stimulate ChR2+ vCA1 pyramidal cell axons while recording from single neurons of the BA in vivo, we implanted a fiber optic above the BA (Figures 2C and S1D). An optical theta burst stimulation (TBS) was delivered during spontaneous non-theta epochs to mimic physiological rhythmic activity at theta frequency (Luo et al., 2011). Optical TBS of vCA1 axons inhibited the firing of BA PNs (n = 10; 2 additional cells were not responsive) during light trains (baseline 0.5 ± 0.05 Hz, light trains 0.1 ± 0.03 Hz, p < 0.0001, n = 10, Figures 2D and 2E), suggesting that the hippocampal input may disynaptically inhibit BA PNs via recruitment of GABAergic INs. Consistent with this hypothesis, the same optogenetic stimulation evoked firing in INs (n = 7; 4 additional cells were not responsive; baseline 1.2 ± 0.2 Hz, light trains 3.4 ± 1 Hz, p < 0.05, Figures 2F and 2G).

To test whether vCA1 axons directly target BA neurons, confocal microscopy of recorded and biocytin-filled BA PNs and INs was used to detect ChR2-YFP-positive boutons apposed to their dendrites (Figures S3A and S3B). Hippocampal axons were found to preferentially innervate PNs by targeting dendritic spines, whereas INs were primarily, but not exclusively, contacted on dendritic shafts (Figure S3E). Further electron microscopy data confirmed that axons of vCA1 pyramidal cells formed excitatory synapses with PNs and INs dendrites (Figures S3C and S3D).

Thus, vCA1 pyramidal cell axons monosynaptically contact PNs and INs of the BA. However, their theta frequency activation evoked opposite responses on PNs and INs recorded in vivo. These effects resembled the contrasting firing rates between PNs and INs observed during theta oscillations.

### TBS of vCA1 Axons Hyperpolarizes PNs via FFI

**Figure 3:**
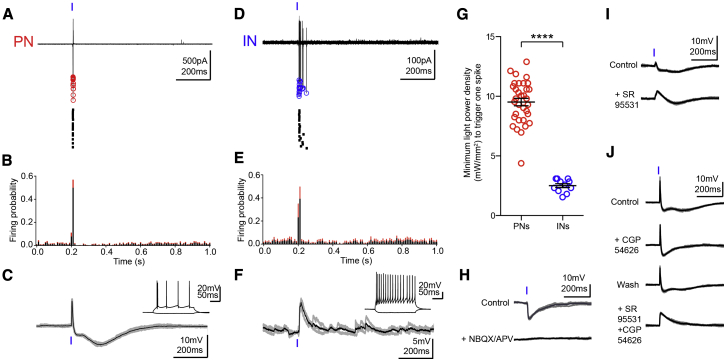
Activation of Hippocampal Inputs Provides FFI of BA PNs (A) Ex vivo cell-attached recording from a representative PN in response to single light pulses (20 superimposed sweeps, top; singularly represented in raster plot, bottom). Red circles denote spike negative peak. (B) Mean probability of firing of PNs (bins = 10 ms, black: mean, red: SEM; n = 24). (C) Whole-cell recording of the same cell as in (A) showing that the light pulse induced a PSP composed of an EPSP followed by a biphasic IPSP. On average, the peak amplitudes of the EPSP, early IPSP, and late IPSP were 8.9 ± 1 mV, 4.1 ± 0.8 mV, and 2.7 ± 0.6 mV (n = 24), respectively. Inset: Response to hyperpolarizing and depolarizing current injections showing stereotypic PN firing. (D) Cell-attached recording from a representative IN in response to a single light pulse (20 superimposed sweeps, top; singularly represented in raster plot, bottom). Blue circles denote spike negative peak. (E) Mean probability of firing of INs (bin = 10 ms, black: mean, red: SEM; n = 11). (F) Whole-cell recording of the same cell as in (D) showing that the light stimulation evoked a monophasic EPSP. On average, the peak amplitude of the EPSP was 8.1 ± 0.7 mV (n = 24). Inset: Response to hyperpolarizing and depolarizing current injections displaying stereotypic IN firing. (G) Minimum light power density (mW/mm^2^) necessary to trigger one spike in PNs (n = 33) and INs (n = 11). Significantly higher power was necessary to reach spike threshold in PNs (^∗∗∗∗^p < 0.0001). (H) The IPSP induced by a single light pulse was blocked by glutamatergic antagonists NBQX (10 μm)/APV (100 μm), suggesting it was mediated by FFI due to activation of BA INs (n = 5). (I) The early IPSP was blocked by the GABA_A_ receptor antagonist SR95531 (10 μm, n = 5). (J) The late IPSP was blocked by the GABA_B_ receptor antagonist CGP54626 (5 μm, n = 26). Co-application of SR95531 and CGP54626 abolished IPSPs and increased the duration of the EPSP. All data from whole-cell recordings shown are three superimposed (gray) and average traces (black). Data are presented as means ± SEM.

Because vCA1 pyramidal cells are glutamatergic, we hypothesized that the inhibition of PNs was due to the disynaptic activation of feedforward INs, while the monosynaptic excitation of PNs would remain subthreshold. To test this hypothesis, we optogenetically dissected the BA circuit activated by vCA1 HPC ex vivo. We prepared acute brain slices 3 to 4 weeks after the ChR2 injection and recorded single BA neurons in cell attached mode (see Supplemental Experimental Procedures). This configuration did not alter the intracellular milieu of the recorded cell and mimicked our extracellular in vivo conditions. PNs and INs could be distinguished according to their soma size (diameter ≥ 20 μm for PNs, < 15 μm for INs). After cell-attached recordings, 8/40 PNs and 6/18 INs were re-patched in whole-cell mode to confirm their identity. Optical stimulations were delivered through the microscope objective to excite vCA1 axons within an area of ∼200 μm diameter around the soma of the recorded neurons. We observed that the power of a single light pulse stimulation had to be significantly higher (p < 0.0001) to trigger one action potential in PNs (9.5 ± 0.3 mW/mm^2^, n = 33) compared to INs (2.5 ± 0.2 mW/mm^2^, n = 11) (Figure 3).

To investigate how synaptic excitation and inhibition evoked by activation of vCA1 fibers influence the firing of BA neurons, we recorded neurons in whole-cell mode. Intracellularly recorded neurons were classified as PNs or INs according to both electrophysiological and anatomical parameters (see Supplemental Experimental Procedures). Single light pulse stimulation triggered a monosynaptic excitatory postsynaptic potential (EPSP) followed by an early-fast and late-slow inhibitory postsynaptic potentials (IPSPs) in 66% of PNs (n = 80/122, Figures 3C and S4A) and EPSP followed by early IPSP in 15% of PNs (n = 18/122, Figure S4B). When we recorded from BA INs (n = 31) or intercalated neurons (ITCs) (n = 13, Figure S5; pooled in the experiments presented below), the same stimulation evoked an EPSP in 61% of the neurons (n = 27/44, Figure 3F). All types of synaptic responses observed in PNs or INs are summarized in the legend of Figure S4. Notably, 11/13 recorded ITCs were histologically verified, and their cell bodies were found either in the external capsule (n = 4), intermediate capsule (n = 4), or main intercalated nucleus (n = 3). The IPSPs were disynaptic because they were abolished by the application of the glutamate receptor antagonists NBQX (10 μM) and APV (100 μM) (Figure 3H, n = 5). In PNs, the early and late IPSPs were mediated by GABA_A_ and GABA_B_ receptors because they were abolished by SR95531 (10 μM, Figures 3I and 3J, n = 5) and CGP54626 (5 μM, Figure 3J, n = 26), respectively. We further confirmed that the optogenetic stimulation activated presynaptic Na^+^ channels by blocking PSPs with 1 μM tetrodotoxin (n = 3, data not shown). Blockade of GABA_A_ receptor alone or together with blockade of GABA_B_ receptors increased the duration of the EPSPs (Figures 3I and 3J), consistently with previous demonstrations that FFI narrows the time window for the integration of synaptic excitation (Gabernet et al., 2005; Pouille and Scanziani, 2001).

**Figure 4:**
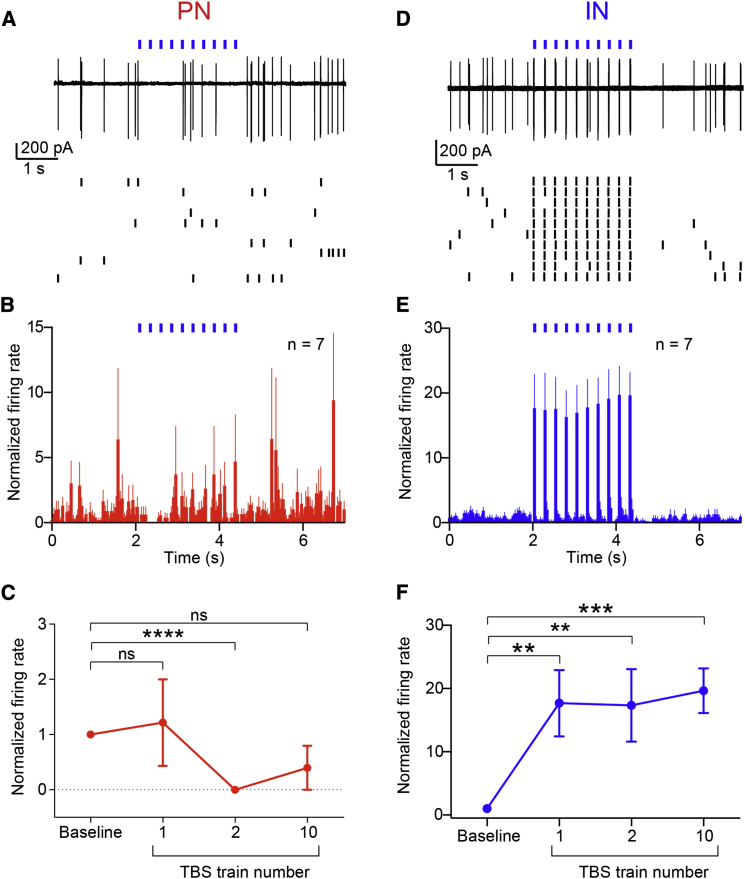
Optical TBS of Hippocampal Axons Transiently Inhibits PNs and Triggers Firing in INs Ex vivo (A) Ex vivo cell-attached recording from a representative BA PN inhibited by TBS of vCA1 axons (ten superimposed sweeps, top; singularly represented in raster plot, bottom). (B) Pooled data showing PNs firing rates over time during TBS (bin = 50 ms, n = 7). (C) Firing rates of PNs compared between baseline, first, second, and tenth trains of the TBS. Firing is significantly reduced during the second train and partially recovers at tenth train. (D) Ex vivo cell-attached recording from a representative BA IN. TBS of vCA1 axons triggers action potentials (ten superimposed sweeps, top; singularly represented in raster plot, bottom). (E) Pooled data showing INs firing rates over time during TBS (bin = 50 ms, n = 7). (F) Firing rates of INs compared between baseline, first, second, and tenth trains of the TBS. Firing is significantly increased during TBS trains. ^∗∗^p < 0.01; ^∗∗∗^p < 0.001; ^∗∗∗∗^p < 0.0001. Data are presented as means ± SEM.

Next, we wished to test whether TBS-evoked inhibition of PNs spontaneous firing occurred in a slice preparation. In these conditions, long-range projections to the BA are cut, ruling out that their stimulation would in turn trigger activation of other regions projecting to the BA. First, we optically activated vCA1 fibers at theta frequency while recording from BA neurons in cell-attached mode, using light intensities sufficient to evoke an action potential in INs but not in PNs (range: 2 to 3 mW/mm^2^). Optogenetic TBS transiently inhibited the firing of PNs (Figures 4A–4C, second train versus baseline p < 0.0001, n = 7). In contrast, TBS triggered action potentials synchronized to each light train in INs (Figures 4D–4F, second train versus baseline p < 0.01, n = 7). We also used higher light intensities, sufficient to evoke action potentials in PNs (>10 mW/mm^2^) to examine the impact of TBS on evoked firing. In this case, optogenetic TBS transiently depressed the evoked spike probability in BA PNs (Figures S6A and S6B, second versus first TBS trains p < 0.001, n = 22) via activation of GABA_B_ receptors (Figures S6E and S6F, second versus first train p > 0.05, n = 9). In contrast, TBS did not reduce the spike probability of INs (Figures S6C and S6D, second versus first TBS trains p > 0.05, n = 10). These results suggest that the activation of hippocampal fibers at theta frequency preferentially recruits GABAergic INs of the BA, inhibiting the spontaneous firing of PNs or sculpting their hippocampal-driven firing via FFI.

### Mechanisms Underlying the Dynamics of TBS of vCA1 Axons in BA Neurons

**Figure 5:**
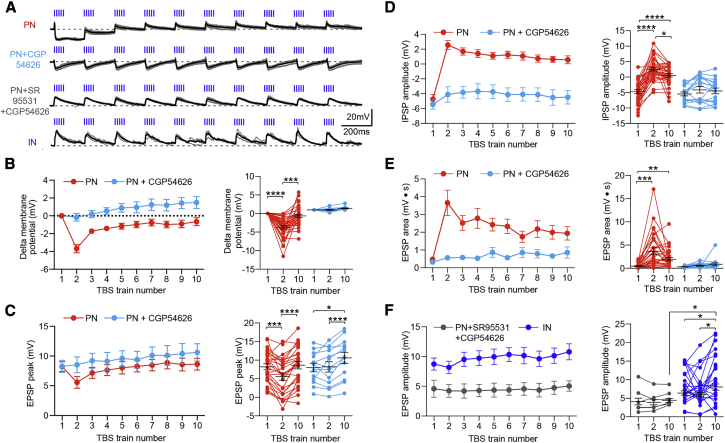
Synaptic Dynamics Evoked by TBS in BA PNs and INs (A) Synaptic potentials (three superimposed sweeps, gray traces; average, black trace) evoked by TBS in representative PNs and in a representative IN in control and in presence of the GABA_A_ (SR95531, 10 μM) and GABA_B_ receptor (CGP54626, 5 μM) antagonists. (B and C) Left, peak of EPSP and membrane potential recorded from PNs for each TBS train in control (n = 34) and in the presence of CGP54626 (n = 17). Right, membrane potential and EPSP peak compared between the first, second, and tenth trains of the TBS. Hyperpolarization and depression of the EPSP peak were blocked by CGP54626. In control, the membrane potential change from baseline was −3.7 ± 0.5 mV at the second train and −0.7 ± 0.5 mV at the tenth train. In the presence of CGP54626, these values were −0.2 ± 0.4 mV and +1.5 ± 0.7 mV. In control, the EPSP peak change from the first train was −2.6 ± 0.6 mV at the second train and +0.4 ± 0.5 mV at the tenth train. In the presence of CGP54626, these values were +0.2 ± 0.7 mV and +2.6 ± 0.8 mV. (D) IPSP amplitude recorded from PNs for each TBS train in control (n = 34) and in presence of CGP54626 (n = 17). Note that the control IPSP that starts as hyperpolarizing (negative potential value) becomes depolarizing in response to the following stimuli during TBS (positive potential values). Right, IPSP amplitude is compared between the first, second, and tenth trains of the TBS. IPSP depression was blocked by CGP54626. In control, the IPSP amplitude change from the first train was +7.3 ± 0.8 mV at the second train and +5.3 ± 0.5 mV at the tenth train. In the presence of CGP54626, these values were +1.3 ± 0.6 mV and +1 ± 0.6 mV. (E) EPSP area recorded from PNs for each TBS train in control (n = 30) and in the presence of CGP54626 (n = 15). Right, broadening of EPSP area with the second TBS train. Broadening still persisted at the tenth train and was blocked by CGP54626. In control, the EPSP area change from the first train was +3.2 ± 0.7 mV at the second train and +1.5 ± 0.4 mV at the tenth train, In the presence of CGP54626, these values were +0.2 ± 0.1 mV and +0.5 ± 0.3 mV. (F) Left, EPSP amplitude for each train of the TBS in presence of SR95531 and CGP54626 in PNs (n = 6) and in INs (n = 23). Right, the EPSP amplitude was stable in PNs in presence of SR95531 and CGP54626. In INs, the EPSP amplitude significantly increased at the tenth TBS train. At the tenth train, EPSP amplitude was significantly more depolarized in INs than PNs. In control, the EPSP amplitude change from the first train was −0.6 ± 1 mV at the second train and +2.6 ± 1 mV at the tenth train. In the presence of CGP54626, these values were −0.3 ± 0.5 mV and +0.5 ± 0.8 mV. ^∗^p < 0.05; ^∗∗^p < 0.01; ^∗∗∗^p < 0.001; ^∗∗∗∗^p < 0.0001. Data are presented as means ± SEM.

Next, we investigated synaptic and membrane excitability dynamics that are likely to cause modulation of firing by TBS in PNs. To this end, we performed whole-cell recordings of PNs and INs in the BA during optical TBS. First, TBS elicited a transient membrane hyperpolarization (Figures 5A–5B, second train versus first train p < 0.0001, n = 34) that resulted in EPSP peak transitorily lowered to more hyperpolarized potentials (Figures 5A and 5C, second train versus first train p < 0.001, n = 34). Both effects were mediated by GABA_B_ receptors, since they were blocked by CGP54626 (5 μM) (Figures 5A–5C, second train versus first train p > 0.05, n = 17). Second, TBS caused a depression of the IPSP amplitude (Figures 5A and 5D, second train versus first train p < 0.0001, n = 34). This led to a significant increase of the EPSP area (Figures 5A and 5E, second train versus first train p < 0.05, n = 30), consistent with FFI constraining the duration of excitatory inputs. These effects were also dependent on GABA_B_ receptor activation, because they were blocked by 5 μM CGP54626 (Figures 5A, 5D, and 5E, second train versus first train p > 0.05, n = 17). Furthermore, when light stimulation elicited only an EPSP followed by GABA_A_-IPSP, as observed in a few PNs (Figure S4B), the membrane hyperpolarization and the depression of IPSP amplitude were greatly reduced (Figure S7, p > 0.05, n = 9), consistent with the GABA_B_ receptor dependency of these effects. In these cases, the GABA reuptake blocker SKF89976A (25 μM) produced a transient hyperpolarization and depression of the IPSP amplitude, most likely through GABA spill-over and activation of extra-synaptic GABA_B_ receptors (Figure S7, n = 6, p < 0.05).

**Figure 6:**
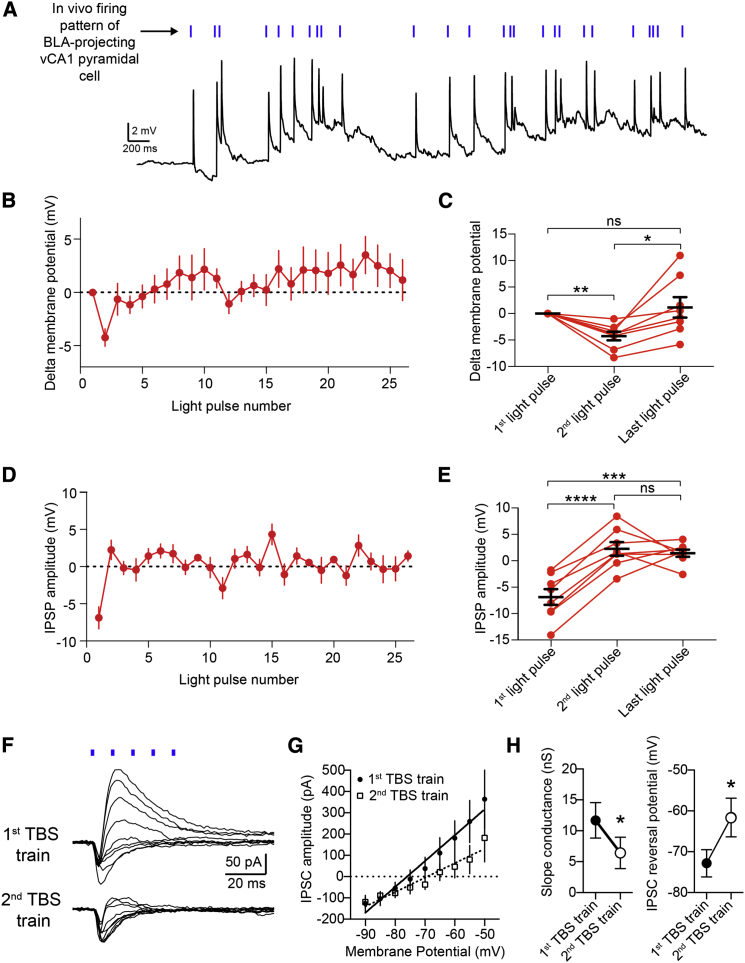
Optical Stimulation of vCA1 Axons with Firing Pattern of a vCA1 Pyramidal Cell Recapitulates Synaptic Dynamics Evoked by TBS (A) Synaptic potentials evoked by optical stimulation of vCA1 axons with firing pattern of a BLA-projecting vCA1 pyramidal cell recorded during exploratory behavior. (B) PNs membrane potential preceding each light pulse (n = 8). (C) Membrane potential compared between first, second, and last light pulses. Hyperpolarization occurs at the second light pulse but is recovered by the last light pulse. Delta membrane potential (compared to membrane potential before the first light pulse) was −4.2 ± 0.8 mV (second light pulse) and +1.2 ± 1.9 mV (last light pulse). (D) IPSP amplitude plotted for each light pulse (n = 8). (E) IPSP amplitude compared between first, second, and last light pulses. IPSP depresses at the second light pulse and remains significantly reduced until the last light pulse. The change of the IPSP amplitude (compared to IPSP amplitude following first light pulse) was +9.1 ± 0.8 mV (second IPSP) and +8.3 ± 1.7 mV (last IPSP). (F) Representative voltage-clamp trace from PN showing TBS-evoked EPSPs and IPSPs at the first and second TBS trains at different holding potentials (from −90 mV to −50 mV, 5-mV steps). (G) IPSC amplitude plotted for each holding potential for first and second TBS trains (n = 6). (H) The second TBS train causes both a significant decrease in GABA_A_ synaptic conductance (left) and a significant shift of the IPSC reversal potential (E_GABA_) toward more depolarized membrane potentials (right). ^∗^p < 0.05; ^∗∗^p < 0.01; ^∗∗∗^p < 0.001; ^∗∗∗∗^p < 0.0001. Data are presented as means ± SEM.

Next, we used as optical stimulation a spike train that was recorded during open field exploration from a vCA1 place cell projecting to the BLA (as well as to the nucleus accumbens and mPFC) (Ciocchi et al., 2015). This stimulation elicited responses in BA PNs highly similar to TBS, namely a transient membrane hyperpolarization (second light pulse versus first light pulse p < 0.001, n = 8) and a dynamic depression of FFI (second light pulse versus first light pulse p < 0.0001, n = 8) (Figures 6A–6E).

Moreover, we tested whether the depression of the IPSP was caused by a reduction in GABA_A_ conductance. We recorded PNs in voltage-clamp mode, applying optical TBS at different holding potentials to plot the peak current of the first and second IPSC versus the holding potential. The slope of this I/V relationship was reduced for the second IPSC (from 11.7 ± 2.9 nS to 6.4 ± 2.6 nS, p < 0.05, n = 6), suggesting that the GABA_A_ conductance was indeed decreased. We also observed that the reversal potential of the IPSC (E_IPSC_) shifted from −72.8 ± 3.3 mV for the first IPSC to −61.6 ± 4.7 mV for second IPSC (p < 0.05, n = 6, Figures 6F–6H). The E_IPSC_ shift suggests that a reduction in the driving force for Cl^−^ contributes to the depression of FFI during TBS, consistent with the residual IPSP depression trend still present with CGP54626 (Figure 5D) and previous reports (Thompson and Gähwiler, 1989; Woodin et al., 2003). Thus, GABA_B_-dependent reduction of GABA_A_ conductance and shift in the E_IPSC_ account for the synaptic dynamics observed.

**Figure 7:**
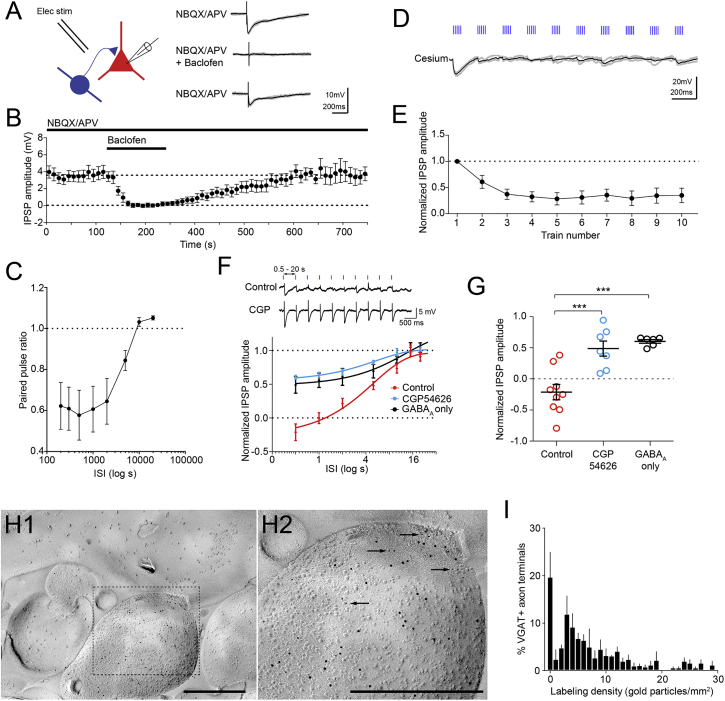
Presynaptic GABA_B_ Receptors at BA IN-PN Synapses Mediate Depression of FFI (A) Left, schematic of the experimental configuration. Local electrical stimulation is applied while recording from PNs. Right, representative current clamp recording (−65 mV) showing monosynaptic IPSP induced by local electrical stimulation in presence of NBQX/APV. The IPSP was abolished by the GABA_B_ receptor agonist baclofen (10 μM). During baclofen application, neurons hyperpolarized but were held at −65 mV to avoid changes in driving force for Cl^−^. (B) Pooled data, (n = 6). (C) PPR of IPSCs recorded in PNs and evoked by local electrical stimulation. PPR reduced with shorter ISIs. (D) The depression of IPSPs during TBS occurred in a PN recorded at 0 mV with cesium-containing intracellular solution. (E) Pooled data (n = 7). (F) Trains of ten single pulses were delivered with variable ISIs (0.5, 1, 2, 5, 10, 15, and 20 s) while recording from PNs. Top, representative trace during which light pulses were delivered at 0.5-s intervals. Depression of the IPSP was largely blocked by CGP54626. Bottom, average of the last three IPSPs evoked for each ISI normalized to the first IPSP. The IPSP amplitude became smaller with shorter ISIs. This depression was reduced when stimulation evoked only GABA_A_ receptor IPSPs or in presence of CGP54626 (5 μM). (G) Averaged amplitudes of last three IPSPs elicited with a train of ten single pulses delivered every 0.5 s: IPSP amplitude in control conditions (n = 9) was significantly lower than in presence of CGP54626 (p < 0.001 versus control, n = 7) or in PNs where only a GABA_A_ IPSP was evoked (p < 0.001 versus control, n = 6). (H1) Electron micrograph of a GABAergic terminal in the BA as obtained with the SDS-digested FRIL method. Scale bar: 500 nm. (H2) Enlarged view of the area within the dashed lines in (H1). Larger gold particles (10 nm) reveal VGAT, whereas smaller gold particles (5 nm; arrowheads) identify GABA_B_ receptors. Scale bar: 500 nm. (I) Frequency distribution of VGAT+ axon terminals with different density of GABA_B_ receptor labeling (n = 179 synapses obtained from three mice). Data are presented as means ± SEM.

The GABA_B_-receptor-mediated effects could be due to the depression of either glutamatergic synapses from vCA1 fibers onto BA INs and/or GABAergic synapses from BA INs onto PNs. We distinguished between these two possibilities by recording directly from GABAergic INs with the soma in the BA and ITCs (Figure S5). The EPSPs recorded in these cells displayed a gradual increase in amplitude through the TBS (Figure 5F, tenth versus first train p < 0.05, n = 23). Hence, vCA1-BA INs excitatory synapses could not be responsible for the transient depression of the IPSPs recorded from PNs. Furthermore, the amplitude of the EPSPs recorded from PNs was stable in presence of antagonists for GABA receptors (Figure 5A, F, tenth versus first train p > 0.05, n = 6), indicating that vCA1-BA PNs excitatory synapses do not undergo depression during TBS. Next, we tested whether presynaptic mechanisms contribute to the depression of the IPSP amplitude. Monosynaptic IPSPs were triggered in PNs through local electrical stimulation in the presence of NBQX (10 μM) and APV (100 μM). The application of the GABA_B_ receptor agonist baclofen (10 μM) abolished the monosynaptic IPSPs (Figures 7A and 7B, n = 5, p < 0.01). The magnitude of this effect suggests that activation of presynaptic GABA_B_ receptors and an increase in postsynaptic membrane conductance induced by baclofen accounted for this effect. To further corroborate the involvement of presynaptic GABA_B_ receptors in the depression of the IPSP amplitude, additional experiments were carried out. First, electrical stimulation was delivered while recording postsynaptic neurons in voltage-clamp mode. The paired pulse ratio (PPR) of electrically evoked inhibitory postsynaptic currents (IPSCs), a parameter critically dependent on presynaptic GABA_B_ receptors (Davies et al., 1990), became smaller with shorter inter-stimuli intervals (ISIs) (Figure 7C, n = 5). Second, we observed depression of IPSPs during optical TBS using a cesium-based intracellular solution to block postsynaptic cesium-sensitive K^+^ channels linked to GABA_B_ receptors (Figures 7D and 7E, n = 7) (Gähwiler and Brown, 1985). Third, we delivered trains of ten single light pulses with varying ISI; the IPSP amplitude was inversely related to the ISIs (Figure 7F). In the presence of 5 μM CGP54626, such use-dependent depression of the IPSPs was significantly reduced (p < 0.001 versus control, Figures 7F and 7G). Likewise, TBS-induced depression of the IPSPs was attenuated in PNs in which GABA_A_-only IPSPs were evoked (p < 0.001 versus control, Figures 7F and 7G). This suggests that when spillover of GABA on extrasynaptic GABA_B_ receptors at postsynaptic site is weak or absent, resulting in GABA_A_ only IPSP, spillover of GABA on presynaptic GABA_B_ receptor (López-Bendito et al., 2004) is also less likely to occur, ensuing more stable IPSPs. Fourth, we detected GABA_B_ receptors at the axon terminals of INs using the SDS-digested freeze-fracture replica immunolabeling (FRIL) method (Figures 7H and 7I). Taken together, these data demonstrate that vCA1-driven FFI on BA PNs is dynamically modulated by GABA_B_ receptors located on the axon terminals of GABAergic INs. What could be the consequences of FFI dynamics for synaptic integration and plasticity in PNs?

### TBS of vCA1 Axons Gates Heterosynaptic Plasticity of LA-BA Excitatory Synapses

**Figure 8:**
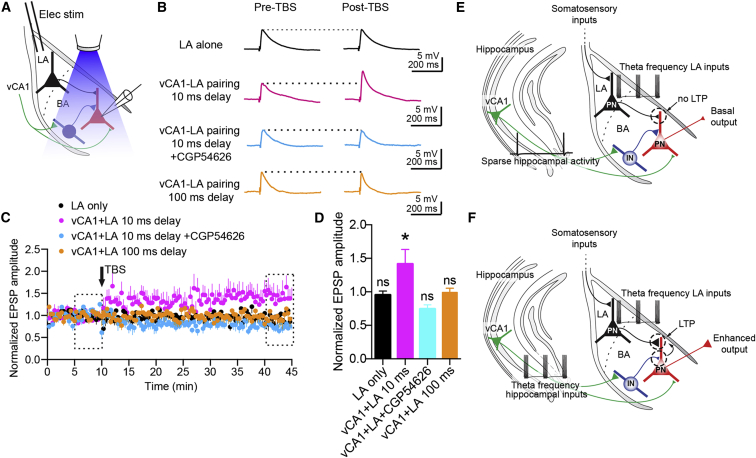
TBS of Hippocampal Inputs Gates LTP of LA-BA Synapses (A) Schematic of the experimental configuration: optical stimulation of vCA1 axons in the BA was paired with electrical stimulation of the LA while recording from PNs. (B) Representative traces (average of ten sweeps) showing EPSPs evoked by electrical stimulation of the LA before and after TBS (50 trains). TBS was applied to the LA only (top), to the LA 10 ms after optical TBS of vCA1 axons without (upper middle) or with CGP54626 (5 μM, lower middle), or to the LA 100 ms after optical TBS of vCA1 axons (bottom). Stimulation artifacts are truncated. (C) The EPSP amplitude plotted over time (stimulation occurs every 10 s). Dashed rectangles represent intervals used for statistical comparisons in (D). Pairing of LA-vCA1 TBS (n = 7) induced LTP, which did not occur with TBS of the LA only (n = 6), but also in the presence of CGP54626 (n = 5) or when TBS of the LA was evoked 100 ms after TBS of the vCA1 axons (n = 7). (D) Quantification of the intervals in (C). Each condition is compared with its own baseline. The EPSP amplitude was significantly increased (^∗^p < 0.05) after LA-vCA1 pairing with 10-ms delay. (E and F) Schematic model of the vCA1-BA circuit. (E) During sparse firing of vCA1 pyramidal neurons projecting to the BA, vCA1 input to BA INs drives FFI onto BA PNs. This results in brief vCA1-mediated excitation and hyperpolarization of PNs via GABA_B_ receptors. Theta frequency stimulation of the LA alone does not elicit LTP. (F) During theta frequency inputs driven by vCA1 pyramidal cells to BA neurons, a GABA_B_-receptor-dependent depression of GABA release from INs occurs. This results in broadening of the temporal window for the integration of excitation in PNs. This mechanism allows the induction of heterosynaptic LTP at LA-BA synapses when LA is simultaneously stimulated at theta frequency. Colored triangles depict presynaptic terminals, and their size indicates synaptic strength. Data are presented as means ± SEM.

The PNs, which are the major output of the BA, represent the downstream target not only of vCA1 axons but also of LA neurons that may convey information from somatosensory areas (Pitkänen et al., 1997). Since the depression of the IPSPs affects the temporal window for synaptic integration, we reasoned that this phenomenon might gate excitatory synaptic plasticity between LA and BA PNs. To test this idea, we electrically stimulated the LA while recording from BA PNs (Figure 8A). First we observed that the monosynaptic EPSP induced by LA stimulation was not significantly different following electrical TBS of the LA (Figures 8B–8D, last 5 min versus baseline p > 0.05, n = 6) even in the presence of the GABA_A_ receptor antagonist picrotoxin (100 μM, last 5 min versus baseline p > 0.05, n = 5, Figures S8A and S8B), ruling out LA-driven FFI as gatekeeper of LA-BA plasticity. Likewise, optical TBS of the vCA1 fibers did not elicit long-term potentiation (LTP) at vCA1-BA synapses either (last 5 min versus baseline p > 0.05, n = 5), but only a short-term potentiation of the EPSPs (Figures S8C and S8D). The latter result is consistent with short-term plasticity evoked by high-frequency electrical stimulation of HPC fibers observed in the BLA in vivo (Maren and Fanselow, 1995). Next, we paired TBS of the LA with optical TBS of vCA1 axons. LA electrical stimuli were delayed by 10 ms from the optical stimuli so that LA inputs occurred near the peak of the IPSP, which depresses during optical TBS. Notably, vCA1-LA pairing induced LTP at LA-BA PNs synapses (Figures 8B–8D, last 5 min versus baseline p < 0.05, n = 7). In contrast, when the LA electrical stimuli were delayed by 100 ms from the optical stimuli, and the vCA1 EPSPs had ended, vCA1-LA pairing did not elicit LTP (Figures 8B–8D, last 5 min versus baseline p > 0.05, n = 7). Finally, the application of 5 μM CGP54626 blocked the pairing-induced LTP (Figures 8B–8D, last 5 min versus baseline p > 0.05, n = 5). Taken together, our data suggest that the vCA1 HPC gates the induction of LTP of LA-BA excitatory connections in a delay-dependent manner by depressing FFI from local INs via presynaptic GABA_B_ receptors (Figures 8E and 8F).

## Discussion

Our data provide learning-relevant cellular mechanisms for hippocampal gating of amygdala information processing. We found that theta frequency firing of vCA1 pyramidal cells caused inhibition of BA PNs firing by driving local INs. Inhibition of PNs was accompanied by a reduction of GABA release from feedforward INs onto PNs via the activation of presynaptic GABA_B_ receptors and by changing postsynaptic GABA_A_-receptor-mediated Cl^−^ driving force. This depression of FFI reduced the hyperpolarization; broadened the temporal window for integration of excitatory stimuli; and, when occurring together with a synchronous input from the LA, gated the induction of LTP at LA-BA synapses.

We found an opposite pattern of activity of BA PNs and INs in vivo following transitions from SWA to theta oscillations in afferent structures (CA1 and TeA). This is consistent with the increase in BLA PNs firing rate during slow-wave sleep (Paré and Gaudreau, 1996). Our data suggest that theta frequency input from vCA1 to the BA may account for this brain-state-dependent firing, as photostimulation of vCA1 axons inhibited BA PNs firing via activation of feedforward INs and GABA_B_ receptors.

One way in which theta frequency activation of afferent vCA1 hippocampal fibers might lead to decreased PN firing is by membrane hyperpolarization. We demonstrate that this was mediated by excitation of feedforward INs and activation of postsynaptic GABA_B_ receptors in PNs. This hyperpolarization implies that sparse excitatory inputs are less effective because they are less likely to reach threshold for spike generation. An explanation of the stronger inhibitory effect of hippocampal theta input on PNs firing observed in vivo might reside in lower PNs firing rates during urethane anesthesia, greater GABA release in an intact brain, and stimulation-driven EPSPs remaining subthreshold. Indeed, higher stimulation intensities were needed to evoke supra-threshold responses in PNs compared to INs in vitro, consistent with previous reports in the cortex (Gabernet et al., 2005). As the number of appositions and, therefore, of putative synaptic contacts made by hippocampal afferents was higher on PNs than on INs, the higher excitability of INs could be explained by greater synaptic strength of vCA1-INs synapses and/or input resistance, which is usually higher in INs than PNs.

Our observations challenge the simple prediction that excitatory inputs from vCA1 would lead to sustained excitation and increase in firing of PNs of the BA. We show that FFI rapidly and powerfully overrides excitation, dampening the spike output of BA PNs. Our data are consistent with prominent FFI triggered by recruitment of INs after the stimulation of excitatory CA1 fibers in the BA (Hübner et al., 2014; Zhang et al., 2014). Moreover, electrical stimulation of the BLA inhibits pyramidal cells and excites INs of the mPFC in anaesthetized rats (Dilgen et al., 2013), suggesting that vHPC, BLA, and mPFC are wired through similar cellular patterns. Hence, it is appealing to speculate that the effect of reduction in firing of putative BLA PNs by directional theta input from the mPFC recently reported (Likhtik et al., 2014) is also mediated by recruitment of local INs.

It is a general principle that FFI shapes synaptic integration in PNs of various brain areas (e.g., Gabernet et al., 2005; Mittmann et al., 2005; Pouille and Scanziani, 2001). In our experiments, activation of vCA1 fibers led to a use- and time-dependent attenuation of FFI of the BA via presynaptic GABA_B_ receptors and by postsynaptic reduction of the IPSC driving force. This triggered a dynamic increase of the temporal window for the integration of coincident EPSPs. As a result, pairing TBS of the LA input with TBS of the vCA1 HPC elicited LTP at LA-BA excitatory synapses onto PNs. Such temporal mechanism of coincidence detection acts in synergy with reduced likelihood to reach the action potential threshold for sparse excitatory inputs caused by membrane potential hyperpolarization. The use-dependent suppression of FFI could also provide a cellular mechanism for the short-lasting facilitation of the EPSP or the field potential evoked in the BLA by optical or electrical high-frequency stimulation of HPC fibers to the BLA (present results and Maren and Fanselow, 1995).

Our data are likely to predict cellular dynamics that underlie physiological interactions between vHPC and LA during behavior. However, because our data have been collected in vivo under anesthesia or in vitro, it is possible that differences exist in awake, behaving animals. Interestingly, we observed similar effects, namely transient membrane hyperpolarization and short-term IPSP plasticity, when a spike train occurring during open field exploration of a vCA1 place cell projecting to the BLA (and also to the nucleus accumbens and mPFC) (Ciocchi et al., 2015) was used as optical stimulation. We speculate that repetitive vCA1 pyramidal cells firing, by broadening the EPSP integration window, can decrease the synchrony at which LA and vCA1 neurons need to fire to lead to EPSP summation and action potential generation in BA PNs. This could increase the likelihood of LA-BA synapses strengthening during behavior, which might be important for associative learning. For example, during fear conditioning somatosensory information reaches the BA primarily via the LA (Pitkänen et al., 1997). Thus, FFI, classically known to sharpen spatial contrast of somatosensory stimuli (Mountcastle and Powell, 1959), could be critical to gate LA information in the BA.

Our data have been obtained using optogenetic, synchronous TBS of several vCA1 hippocampal fibers. Such synchronous activation has been used to mimic hippocampal rhythmic theta oscillations (Luo et al., 2011). The exposure to a single behavioral context consistently activates an elevated number of CA1 cells in the mouse (Liu et al., 2014), as it occurs in the TBS protocol where several CA1 fibers are activated by the optogenetic stimulation. It is important to keep in mind, however, that CA1 pyramidal cells usually do not fire simultaneously (e.g., Pastalkova et al., 2008) and that the number of active place cells is lower in vHPC than in dHPC (Kjelstrup et al., 2008; Royer et al., 2010). Furthermore, the stimulation of vCA1 fibers would activate not only the feedforward circuit defined here, but also other parallel circuits. These could include (1) activation of feedback INs that would in turn inhibit PNs, (2) disynaptic excitation of PNs by axon collaterals of nearby PNs, and (3) antidromic activation of vCA1 pyramidal cells leading to release of glutamate in other brain areas, such as mPFC, in turn projecting to the BA. Regarding the latter, individual vCA1 neurons can project to both the mPFC and the amygdala (Ciocchi et al., 2015; Ishikawa and Nakamura, 2006), as well as to other brain structures (Arszovszki et al., 2014; Ciocchi et al., 2015). However, we observed modulation of firing patterns by TBS of vCA1 fibers in neurons recorded in vitro. In these conditions, changes in the excitability exerted by other extra-amygdaloid inputs, such as those from the mPFC should be negligible.

Our data further suggest that activation of GABA_B_ receptors plays a crucial role in the induction of long-term plasticity at BA synapses. Specifically, we propose that vCA1 theta inputs gate LTP of LA-BA synapses by depressing FFI from local INs via GABA_B_ receptors located at their axon terminals. This mechanism occurring in BA neurons mirrors the suppression of FFI by dopamine, gating the induction of LTP in the LA (Bissière et al., 2003). While GABA_B_ receptors at excitatory fibers suppress TBS-induced LTP in LA PNs (Pan et al., 2009), our data demonstrate that GABA_B_ receptors at inhibitory axon terminals promote theta-burst-induced LTP in BA PNs. We cannot exclude that other mechanisms in addition to FFI depression could be involved in the heterosynaptic plasticity we observed. Among them, we document the significant shift toward more depolarized membrane potentials of the E_IPSC_. Moreover, the activation of parvalbumin (PV)+ INs by vCA1 input could in turn inhibit somatostatin-expressing INs, leading to disinhibition of the dendrites of PNs (Wolff et al., 2014). In this regard, a recent report suggests the activation of several types of BA INs, including fast-spiking and non-fast spiking PV+ cells, as well as PV-negative INs by CA1 fibers in the mouse (Hübner et al., 2014). Furthermore, we report here that ITCs show excitatory responses to the optogenetic stimulation of vCA1 axon in the BA. This result is consistent with anatomical data obtained with classical tracing methods in the rat (Canteras and Swanson, 1992; Kishi et al., 2006). In addition to ITCs of the external capsule (Marowsky et al., 2005), it is likely that some ITCs with the soma in the intermediate capsule also mediate FFI of BA PNs (Asede et al., 2015; Bienvenu et al., 2015).

Strengthening of LA-BA excitatory synapses, which are critical for the somatosensory information flow to the BA, could imply stronger BA excitatory output. This could have repercussions on central amygdala neurons and extra-amygdaloid areas targeted by these projection neurons to modulate fear responses. Future studies could clarify whether an optogenetic hippocampal theta input to the BA can alter fear learning. Facilitation of LTP at LA-BA synapses suggests that a theta frequency vCA1 input to the BA could strengthen fear learning. This hypothesis is appealing because “fear neurons” receive prominent inputs from vHPC (Herry et al., 2008), but further experimental evidence is necessary to validate it.

In summary, our findings provide critical cellular and molecular mechanisms mediating vHPC-amygdala interactions. Dissection of this circuit is essential not only to decode fear memory formation, but also to unravel the cellular basis of psychiatric disorders whereby HPC-amygdala changes have been reported (Ghosh et al., 2013; Santos et al., 2013; Zhang et al., 2014).

## Experimental Procedures

### Animals

CaMKIIα-Cre+/+ (Jackson Laboratories, B6.Cg-Tg(Camk2a-cre)T29-1Stl/J, stock number 005359) and wild-type C57BL/6J mice (Charles River) were housed with their littermates with ad libitum access to food and water in a dedicated housing room with a 12-/12-hr light/dark cycle. All procedures involving experimental animals were performed in compliance with the European Convention for the Protection of Vertebrate Animals used for Experimental and Other Scientific Purposes (ETS no. 123); the European Communities Council Directive of 24 November 1986 (86/609/EEC); the Animals (Scientific Procedures) Act, 1986 (UK); and associated regulations under approved project license by Home Office UK (30/2539), the Austrian Animal Experimentation Ethics Board (GZ66.011/28-BrGT/2009), and with Society for Neuroscience Policies on the Use of Animals in Neuroscience Research.

### Expression of ChR2 in vCA1 Pyramidal Neurons

CaMKIIα-Cre mice (P20-P25) were anesthetized using 0.5%–2% isoflurane in oxygen (2 l/min). Analgesia was provided (buprenorphine, 0.1 mg/kg of body weight). In order to selectively express ChR2 in pyramidal neurons, the Cre-inducible recombinant viral vector AAV-EF1a-DIO-hChR2(E123T/T159C)-EYFP (UNC vector core, 0.3 μl volume) was delivered in the right ventral CA1/intermediate CA1 (cited as vCA1 in the text, 3 mm posterior, +3.5 lateral from bregma and 3 mm ventral from the brain surface) through a glass pipette at a rate of 0.1 μl/min. At least 3 weeks were allowed for ChR2 expression before recording procedures. In preliminary experiments, we also used AAV-EF1a-DIO-hChR2(H134R)-EYFP. However, this vector was not used in any subsequent experiment because of its slower kinetics (see Figure S2). Single and high-frequency light pulses reliably evoked action potentials recorded from the soma of vCA1 pyramidal cells expressing AAV-EF1a-DIO-hChR2(E123T/T159C)-EYFP (Figures S2D and S2E). However, we also found that the half-width of the EPSC evoked by a burst (100 Hz) or by a single optical stimulation did not differ (Figures S2F and S2G, p > 0.05, n = 5). Furthermore, the EPSCs were not detectable after the first stimulation for each burst at 100 Hz and depressed for bursts with longer inter-event intervals (Figures S2H and S2I). This strong synaptic depression could either represent a physiological effect at vCA1-BA synapses or be mediated by the kinetics of the activation of axonal ChR2. Although E123T/T159C ChR2 could fire vCA1 pyramidal cells at 100 Hz, effects at the axon terminal may be different (for similar different kinetics of ChR2 at soma versus axon terminal, see Jackman et al., 2014). It is important to note that stimulations at 5–10 Hz (theta frequency) evoked EPSCs that only weakly depressed (Figure S2I).

### In Vivo Recordings, Photostimulation, and Analysis

Surgical procedures and in vivo extracellular recordings and juxtacellular recordings/labeling were performed and analyzed as described in Bienvenu et al. (2012), with minor modifications. Mice were anesthetized with intraperitoneal injections of urethane (1.5 g/kg body weight). To monitor the firing of amygdala neurons in relation to hippocampal SWA and theta oscillations, extracellular recordings were performed from the BA and the dorsal CA1 using glass pipettes filled with 3% Neurobiotin (Vector Laboratories) in 0.5 M NaCl (10–18 MΩ resistance in situ). To photostimulate vCA1 pyramidal cell axons innervating the BA in vivo, a fiber optic cannula connected to a Luxx 488-200 diode laser (Omicron) was implanted 0.5–0.7 mm above the recording site (Figure S1D). TBS consisted of 50 trains of five light pulses (3 ms duration) delivered at 10-ms intervals (100 Hz), with 200 ms between trains. TBS was delivered every 20 s and was repeated for 8–15 trials for each neuron. Data were analyzed off-line using Spike2 built-in functions and MATLAB (Mathworks, Inc.) custom scripts. Full details on in vivo electrophysiology, optogenetics, and analysis are given in the Supplemental Information.

### Ex Vivo Recordings, Photostimulation, and Analysis

Preparation and maintenance of acute slices in vitro and composition of extracellular and intracellular solutions for electrophysiological experiments were performed according to previously published procedures (Mańko et al., 2012) and as described in Alcami et al. (2012), with minor modifications. Optical stimulation of hippocampal ChR2-expressing afferents in the BA in vitro was performed using an optoLED system (Cairn Research) mounted on a Zeiss Axioskop 2 FS microscope. The spot size corresponded to an area of about 200 μm. Optical TBS consisted of ten trains delivered at the same frequency as for the in vivo experiments. Analysis of electrophysiological signals was performed using MATLAB custom scripts. Full details on ex vivo electrophysiology, optogenetics, and analysis are given in the Supplemental Information.

### Statistical Testing

Data are presented as means ± SEM. For electrophysiological data, distributions passing Shapiro-Wilk test for normality were compared using Student’s t tests. Non-Gaussian distributions were compared using non-parametric tests (Mann-Whitney U test, Wilcoxon signed rank test). Comparisons of individual TBS trains were performed with ANOVAs and Bonferroni post hoc tests. Differences were considered significant at p < 0.05. For anatomical data, distributions were compared with Mann-Whitney U test. Differences were considered significant at p < 0.001.

### Drugs and Chemicals

All drugs were obtained from Tocris Biosciences and Sigma-Aldrich.

### Tissue Processing and Anatomical Analyses

Details on brain and slice fixation, immunocytochemistry, confocal, and electron microscopy (including FRIL) are given in the Supplemental Information.

## Author Contributions

Designed experiments: M. Bazelot, M. Bocchio, M.C., and F.F.; Performed experiments: M. Bazelot, M. Bocchio, Y.K., and D.F.; Analyzed data: M. Bazelot, M. Bocchio, Y.K., and D.F.; Supervised the project: M.C., F.F., and P.D.D.; Wrote the paper: M.C. and M. Bocchio.

## Supplemental Information

Document S1. Figures S1–S8 and Supplemental Experimental Procedures

Document S2. Article plus Supplemental Information

## References

[bib1] Alcami P., Franconville R., Llano I., Marty A. (2012). Measuring the firing rate of high-resistance neurons with cell-attached recording. J. Neurosci..

[bib2] Arszovszki A., Borhegyi Z., Klausberger T. (2014). Three axonal projection routes of individual pyramidal cells in the ventral CA1 hippocampus. Front. Neuroanat..

[bib3] Asede D., Bosch D., Lüthi A., Ferraguti F., Ehrlich I. (2015). Intercalated cells provide fear-learning modulated sensory feedforward and feedback inhibition to the basolateral amygdala. Neuron.

[bib4] Berndt A., Schoenenberger P., Mattis J., Tye K.M., Deisseroth K., Hegemann P., Oertner T.G. (2011). High-efficiency channelrhodopsins for fast neuronal stimulation at low light levels. Proc. Natl. Acad. Sci. USA.

[bib5] Bienvenu T.C., Busti D., Magill P.J., Ferraguti F., Capogna M. (2012). Cell-type-specific recruitment of amygdala interneurons to hippocampal theta rhythm and noxious stimuli in vivo. Neuron.

[bib6] Bienvenu T.C., Busti D., Micklem B.R., Mansouri M., Magill P.J., Ferraguti F., Capogna M. (2015). Large intercalated neurons of amygdala relay noxious sensory information. J. Neurosci..

[bib7] Bissière S., Humeau Y., Lüthi A. (2003). Dopamine gates LTP induction in lateral amygdala by suppressing feedforward inhibition. Nat. Neurosci..

[bib8] Canteras N.S., Swanson L.W. (1992). Projections of the ventral subiculum to the amygdala, septum, and hypothalamus: a PHAL anterograde tract-tracing study in the rat. J. Comp. Neurol..

[bib9] Ciocchi S., Passecker J., Malagon-Vina H., Mikus N., Klausberger T. (2015). Brain computation. Selective information routing by ventral hippocampal CA1 projection neurons. Science.

[bib10] Davies C.H., Davies S.N., Collingridge G.L. (1990). Paired-pulse depression of monosynaptic GABA-mediated inhibitory postsynaptic responses in rat hippocampus. J. Physiol..

[bib11] Dilgen J., Tejeda H.A., O’Donnell P. (2013). Amygdala inputs drive feedforward inhibition in the medial prefrontal cortex. J. Neurophysiol..

[bib12] Fanselow M.S. (2010). From contextual fear to a dynamic view of memory systems. Trends Cogn. Sci..

[bib13] Felix-Ortiz A.C., Tye K.M. (2014). Amygdala inputs to the ventral hippocampus bidirectionally modulate social behavior. J. Neurosci..

[bib14] Felix-Ortiz A.C., Beyeler A., Seo C., Leppla C.A., Wildes C.P., Tye K.M. (2013). BLA to vHPC inputs modulate anxiety-related behaviors. Neuron.

[bib15] Gabernet L., Jadhav S.P., Feldman D.E., Carandini M., Scanziani M. (2005). Somatosensory integration controlled by dynamic thalamocortical feed-forward inhibition. Neuron.

[bib16] Gähwiler B.H., Brown D.A. (1985). GABAB-receptor-activated K+ current in voltage-clamped CA3 pyramidal cells in hippocampal cultures. Proc. Natl. Acad. Sci. USA.

[bib17] Ghosh S., Laxmi T.R., Chattarji S. (2013). Functional connectivity from the amygdala to the hippocampus grows stronger after stress. J. Neurosci..

[bib18] Goshen I., Brodsky M., Prakash R., Wallace J., Gradinaru V., Ramakrishnan C., Deisseroth K. (2011). Dynamics of retrieval strategies for remote memories. Cell.

[bib19] Herry C., Ciocchi S., Senn V., Demmou L., Müller C., Lüthi A. (2008). Switching on and off fear by distinct neuronal circuits. Nature.

[bib20] Hübner C., Bosch D., Gall A., Lüthi A., Ehrlich I. (2014). Ex vivo dissection of optogenetically activated mPFC and hippocampal inputs to neurons in the basolateral amygdala: implications for fear and emotional memory. Front. Behav. Neurosci..

[bib21] Ishikawa A., Nakamura S. (2006). Ventral hippocampal neurons project axons simultaneously to the medial prefrontal cortex and amygdala in the rat. J. Neurophysiol..

[bib22] Jackman S.L., Beneduce B.M., Drew I.R., Regehr W.G. (2014). Achieving high-frequency optical control of synaptic transmission. J. Neurosci..

[bib23] Jin J., Maren S. (2015). Fear renewal preferentially activates ventral hippocampal neurons projecting to both amygdala and prefrontal cortex in rats. Sci. Rep..

[bib24] Kishi T., Tsumori T., Yokota S., Yasui Y. (2006). Topographical projection from the hippocampal formation to the amygdala: a combined anterograde and retrograde tracing study in the rat. J. Comp. Neurol..

[bib25] Kjelstrup K.B., Solstad T., Brun V.H., Hafting T., Leutgeb S., Witter M.P., Moser E.I., Moser M.B. (2008). Finite scale of spatial representation in the hippocampus. Science.

[bib26] Lesting J., Narayanan R.T., Kluge C., Sangha S., Seidenbecher T., Pape H.C. (2011). Patterns of coupled theta activity in amygdala-hippocampal-prefrontal cortical circuits during fear extinction. PLoS ONE.

[bib27] Likhtik E., Stujenske J.M., Topiwala M.A., Harris A.Z., Gordon J.A. (2014). Prefrontal entrainment of amygdala activity signals safety in learned fear and innate anxiety. Nat. Neurosci..

[bib28] Liu X., Ramirez S., Tonegawa S. (2014). Inception of a false memory by optogenetic manipulation of a hippocampal memory engram. Philos. Trans. R. Soc. Lond. B Biol. Sci..

[bib29] López-Bendito G., Shigemoto R., Kulik A., Vida I., Fairén A., Luján R. (2004). Distribution of metabotropic GABA receptor subunits GABAB1a/b and GABAB2 in the rat hippocampus during prenatal and postnatal development. Hippocampus.

[bib30] Luo A.H., Tahsili-Fahadan P., Wise R.A., Lupica C.R., Aston-Jones G. (2011). Linking context with reward: a functional circuit from hippocampal CA3 to ventral tegmental area. Science.

[bib31] Mańko M., Bienvenu T.C., Dalezios Y., Capogna M. (2012). Neurogliaform cells of amygdala: a source of slow phasic inhibition in the basolateral complex. J. Physiol..

[bib32] Maren S., Fanselow M.S. (1995). Synaptic plasticity in the basolateral amygdala induced by hippocampal formation stimulation in vivo. J. Neurosci..

[bib33] Maren S., Quirk G.J. (2004). Neuronal signalling of fear memory. Nat. Rev. Neurosci..

[bib34] Maren S., Phan K.L., Liberzon I. (2013). The contextual brain: implications for fear conditioning, extinction and psychopathology. Nat. Rev. Neurosci..

[bib35] Marowsky A., Yanagawa Y., Obata K., Vogt K.E. (2005). A specialized subclass of interneurons mediates dopaminergic facilitation of amygdala function. Neuron.

[bib36] Mittmann W., Koch U., Häusser M. (2005). Feed-forward inhibition shapes the spike output of cerebellar Purkinje cells. J. Physiol..

[bib37] Mountcastle V.B., Powell T.P. (1959). Neural mechanisms subserving cutaneous sensibility, with special reference to the role of afferent inhibition in sensory perception and discrimination. Bull. Johns Hopkins Hosp..

[bib38] Müller M., Faber-Zuschratter H., Yanagawa Y., Stork O., Schwegler H., Linke R. (2012). Synaptology of ventral CA1 and subiculum projections to the basomedial nucleus of the amygdala in the mouse: relation to GABAergic interneurons. Brain Struct. Funct..

[bib39] Orsini C.A., Kim J.H., Knapska E., Maren S. (2011). Hippocampal and prefrontal projections to the basal amygdala mediate contextual regulation of fear after extinction. J. Neurosci..

[bib40] Pan B.X., Dong Y., Ito W., Yanagawa Y., Shigemoto R., Morozov A. (2009). Selective gating of glutamatergic inputs to excitatory neurons of amygdala by presynaptic GABAb receptor. Neuron.

[bib41] Paré D., Gaudreau H. (1996). Projection cells and interneurons of the lateral and basolateral amygdala: distinct firing patterns and differential relation to theta and delta rhythms in conscious cats. J. Neurosci..

[bib42] Paré D., Collins D.R., Pelletier J.G. (2002). Amygdala oscillations and the consolidation of emotional memories. Trends Cogn. Sci..

[bib43] Pastalkova E., Itskov V., Amarasingham A., Buzsáki G. (2008). Internally generated cell assembly sequences in the rat hippocampus. Science.

[bib44] Phelps E.A. (2004). Human emotion and memory: interactions of the amygdala and hippocampal complex. Curr. Opin. Neurobiol..

[bib45] Pitkänen A., Savander V., LeDoux J.E. (1997). Organization of intra-amygdaloid circuitries in the rat: an emerging framework for understanding functions of the amygdala. Trends Neurosci..

[bib46] Pitkänen A., Pikkarainen M., Nurminen N., Ylinen A. (2000). Reciprocal connections between the amygdala and the hippocampal formation, perirhinal cortex, and postrhinal cortex in rat. A review. Ann. N Y Acad. Sci..

[bib47] Popa D., Duvarci S., Popescu A.T., Léna C., Paré D. (2010). Coherent amygdalocortical theta promotes fear memory consolidation during paradoxical sleep. Proc. Natl. Acad. Sci. USA.

[bib48] Pouille F., Scanziani M. (2001). Enforcement of temporal fidelity in pyramidal cells by somatic feed-forward inhibition. Science.

[bib49] Richardson M.P., Strange B.A., Dolan R.J. (2004). Encoding of emotional memories depends on amygdala and hippocampus and their interactions. Nat. Neurosci..

[bib50] Royer S., Sirota A., Patel J., Buzsáki G. (2010). Distinct representations and theta dynamics in dorsal and ventral hippocampus. J. Neurosci..

[bib51] Santos M., D’Amico D., Spadoni O., Amador-Arjona A., Stork O., Dierssen M. (2013). Hippocampal hyperexcitability underlies enhanced fear memories in TgNTRK3, a panic disorder mouse model. J. Neurosci..

[bib52] Seidenbecher T., Laxmi T.R., Stork O., Pape H.C. (2003). Amygdalar and hippocampal theta rhythm synchronization during fear memory retrieval. Science.

[bib53] Thompson S.M., Gähwiler B.H. (1989). Activity-dependent disinhibition. I. Repetitive stimulation reduces IPSP driving force and conductance in the hippocampus in vitro. J. Neurophysiol..

[bib54] Wolff S.B., Gründemann J., Tovote P., Krabbe S., Jacobson G.A., Müller C., Herry C., Ehrlich I., Friedrich R.W., Letzkus J.J., Lüthi A. (2014). Amygdala interneuron subtypes control fear learning through disinhibition. Nature.

[bib55] Woodin M.A., Ganguly K., Poo M.M. (2003). Coincident pre- and postsynaptic activity modifies GABAergic synapses by postsynaptic changes in Cl- transporter activity. Neuron.

[bib56] Zhang C.L., Houbaert X., Lepleux M., Deshors M., Normand E., Gambino F., Herzog E., Humeau Y. (2014). The hippocampo-amygdala control of contextual fear expression is affected in a model of intellectual disability. Brain Struct. Funct..

