# Supplementary figures and images for: Hippocampal Theta Input to the Amygdala Shapes Feedforward Inhibition to Gate Heterosynaptic Plasticity
Source: Neuron. 2015 Sep 23;87(6):1290–303. doi: 10.1016/j.neuron.2015.08.024 (PMC4590554; doi:10.1016/j.neuron.2015.08.024)

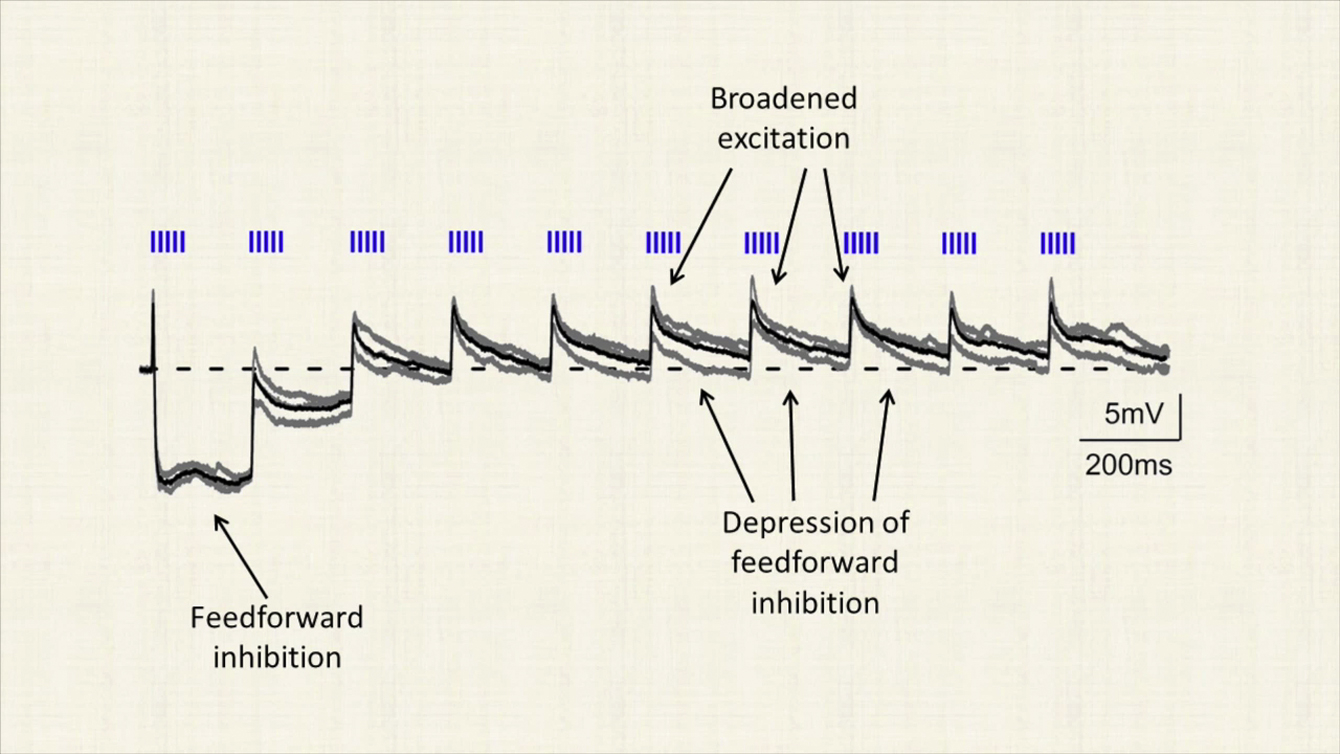

Supplement: Supplementary file 1 [file mmc3.jpg]
