# Supplementary material for: Hippocampal Theta Input to the Amygdala Shapes Feedforward Inhibition to Gate Heterosynaptic Plasticity
Source: Neuron. 2015 Sep 23;87(6):1290–303. doi: 10.1016/j.neuron.2015.08.024 (PMC4590554; doi:10.1016/j.neuron.2015.08.024)
Supplement: Document S1. Figures S1–S8 and Supplemental Experimental Procedures [file mmc1.pdf]

Neuron, Volume *87*

Supplemental Information

Hippocampal Theta Input to the Amygdala

Shapes Feedforward Inhibition

to Gate Heterosynaptic Plasticity

Michaël Bazelot, Marco Bocchio, Yu Kasugai, David Fischer, Paul D. Dodson, Francesco Ferraguti, and Marco Capogna

SUPPLEMENTAL INFORMATION

Hippocampal theta input to the amygdala shapes feedforward inhibition to gate heterosynaptic plasticity

Michaël Bazelot*, Marco Bocchio*, Yu Kasugai, David Fischer, Paul D. Dodson, Francesco Ferraguti & Marco Capogna

INVENTORY OF SUPPLEMENTAL INFORMATION

Contains 8 Supplemental Figures, Supplemental Experimental Procedures, and Supplemental References

- **Fig. S1, related to Fig. 1 and 2**: Clustering of PNs and INs and histologically verified positions of fiber optic tip and recorded neurons. This figure illustrates the different distribution of firing characteristics in PNs and INs, and the locations of the fiber optics and recorded neurons.

- **Fig. S2, related to Fig. 2-8**: Responses to high frequency stimulation of vCA1 pyramidal cell soma or axons expressing ultrafast ChR2/YFP. This figure compares the kinetics of wild type and ultra-fast ChR2.

- **Fig. S3, related to Fig. 2-8:** CA1 pyramidal cells axons form asymmetric synapses onto both INs and PNs of the BA. This figure demonstrates monosynaptic inputs from vCA1 fibers onto BA PNs and INs.

- **Fig. S4, related to Fig. 3, 5 and 7**: Optogenetic stimulation of vCA1 axons evokes heterogeneous responses in BA PNs. This figure illustrates types of synaptic responses evoked in BA PNs by single light stimulation of vCA1 fibers.

- **Fig. S5, related to Fig. 3 and 5**: ITC neurons respond to vCA1 stimulation. This figure shows that ITC neurons receive excitatory input from vCA1.

- **Fig. S6, related to Fig. 4**: High intensity TBS of vCA1 axons transiently depresses the firing of PNs in a $GABA_B$ receptor manner, and reliably activates INs *ex vivo*. This figure shows that feedforward inhibition sculptures vCA1-evoked firing of PNs in a $GABA_B$ receptor-dependent manner.

- **Fig. S7, related to Fig. 5 and 7**: Blockade of GABA re-uptake evokes depression of FFI in PNs with vCA1-evoked $GABA_A$ receptor only IPSPs. This figure demonstrates that GABA spillover promotes presynaptic $GABA_B$ receptor-mediated depression of feedforward inhibition.

- **Fig. S8, related to Fig. 8:** Blockade of $GABA_A$ receptors does not unmask LA-BA PN LTP, and TBS of vCA1 axons does not evoke vCA1-BA PN LTP. This figure rules out the occurrence of LA-BA LTP when inhibition is pharmacologically blocked and the occurrence of vCA1-BA LTP.

Supplemental Experimental Procedures describe electrophsiological, optogenetic and anatomical methods.

Fig. S1

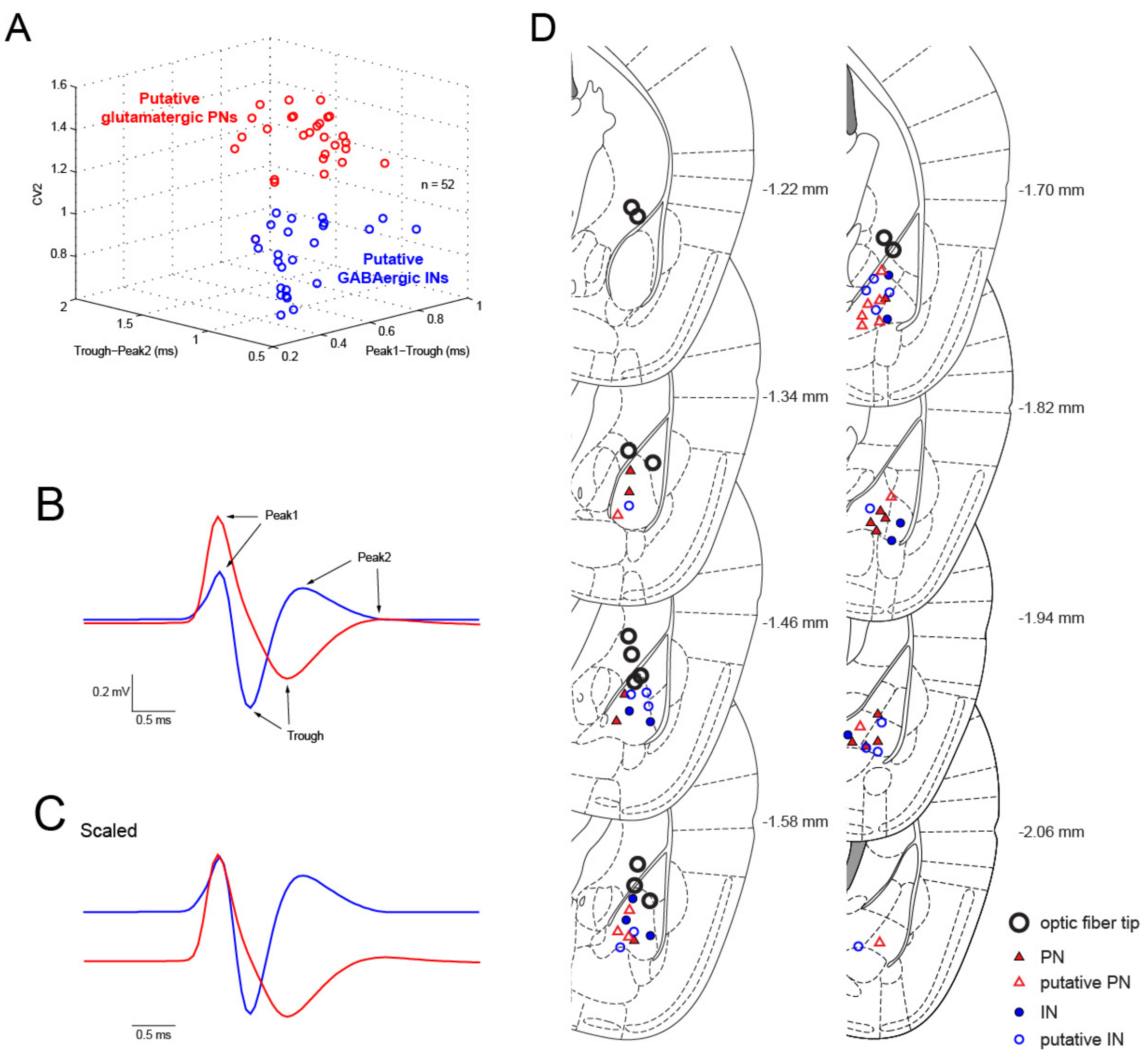

A
Putative glutamatergic PNs
Putative GABAergic INs
n = 52
CV2
Trough−Peak2 (ms)
Peak1−Trough (ms)
B
Peak1
Peak2
Trough
0.2 mV
0.5 ms
C
Scaled
0.5 ms
D
-1.22 mm
-1.34 mm
-1.46 mm
-1.58 mm
-1.70 mm
-1.82 mm
-1.94 mm
-2.06 mm
optic fiber tip
PN
putative PN
IN
putative IN

Fig. S1, related to Fig. 1-2

Clustering of PNs and INs and histologically verified positions of fiber optic tip and recorded neurons.

A, recorded neurons (n = 52) could be separated into putative principal neurons (PNs, n = 27) and interneurons (INs, n = 25) of the mouse basal amygdala (BA) using *K-means* clustering on the basis of three parameters: mean CV2 of the interspike interval (an index of regularity), time between first action potential peak and trough, time between action potential trough and second peak. **B**, averages of 100 spikes of a putative PN (red) and a putative IN (blue) illustrating the methodology used for quantification. **C**, scaled averages of the action potential in B. **D**, histologically verified positions of the tip of fiber optic cannulas (black empty circles, each one represents a separate experiment, n = 13) and *in vivo* recorded neurons. Juxtacellularly labeled and immunohistochemically verified neurons are represented with filled red (PNs) and blue (INs) shapes. Approximate positions of unlabeled (putative) neurons (empty red and blue shapes) were calculated using a labeled reference neuron in the same brain. The fiber optic cannula was implanted from the front with a 16° angle (see Experimental Procedures).

Fig. S2

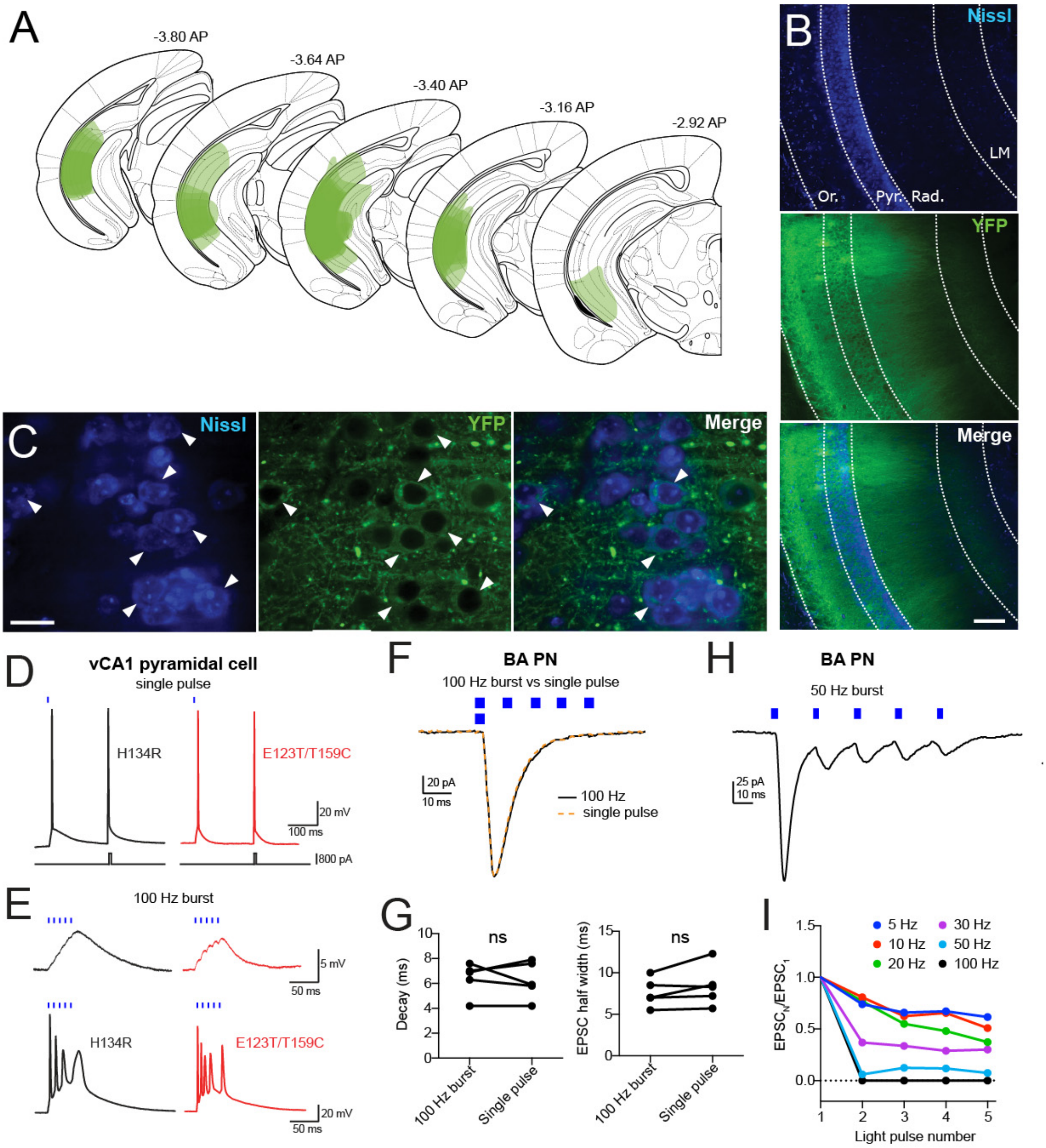
A
-3.80 AP
-3.64 AP
-3.40 AP
-3.16 AP
-2.92 AP
B
Nissl
LM
Or.
Pyr.
Rad.
YFP
Merge
C
Nissl
YFP
Merge
D
vCA1 pyramidal cell
single pulse
H134R
E123T/T159C
20 mV
100 ms
800 pA
E
100 Hz burst
5 mV
50 ms
H134R
E123T/T159C
20 mV
50 ms
F
BA PN
100 Hz burst vs single pulse
20 pA
10 ms
100 Hz
single pulse
G
ns
Decay (ms)
100 Hz burst
Single pulse
ns
EPSC half width (ms)
100 Hz burst
Single pulse
H
BA PN
50 Hz burst
25 pA
10 ms
I
5 Hz
10 Hz
20 Hz
30 Hz
50 Hz
100 Hz
EPSC_N/EPSC_1
Light pulse number

Fig. S2, related to Fig. 2-8

Responses to high frequency stimulation of vCA1 pyramidal cell soma or axons expressing ultrafast ChR2/YFP.

A, localization of 32 injection sites restricted to the vCA1/intermediate CA1 areas. Color intensity reflects the frequency of overlapping injection sites. **B**, Nissl staining of the vCA1 pyramidal cell layer and yellow fluorescent protein (YFP)-Channelrhodopsin-2 (ChR2) staining labeling vCA1 pyramidal cells. Scale bar: 200 µm. **C,** high magnification showing expression of YFP-ChR2 in the membrane of individual pyramidal cell somata (arrowheads). Scale bar: 20 µm. **D**, action potentials elicited by a single light pulse (470 nm, 0.1 ms, left) and by a current step (+800 pA, 0.9 ms) in vCA1 pyramidal cells infected either with H134R (black traces) or E123T/T159C-ChR2 (red traces). After-depolarization recorded in neurons transfected with E123T/T159C-ChR2 showed faster decay with a similar kinetics that the one induced by a short current pulse. **E**, 100 Hz burst stimulation (5 light pulses, 1 ms duration), as used in theta burst stimulation (TBS), produces a depolarization with faster kinetic and a more reliable induction of action potentials in vCA1 pyramidal cells when the E123T/T159C-ChR2 is used (compare red with black traces, n = 3). *Top*, subthreshold response (low light intensity). *Bottom*, suprathreshold response (higher light intensity). **F**, representative superimposed EPSCs recorded in voltage clamp from a BA PN (-75 mV) evoked either by 100 Hz burst stimulation (5 light pulses, 3 ms duration, 2 mW/mm^2) or by a single light pulse (3 ms duration, 2 mW/mm^2). **G,** decay and half-width of burst-evoked and single pulse-evoked excitatory postsynaptic currents (EPSCs) are similar (both $p > 0.05$, n = 5). **H**, representative EPSCs recorded from a BA PN (-75 mV) evoked by a 50 Hz burst stimulation. **I,** EPSCs amplitude (normalized to the 1st EPSC) evoked by 5-100 Hz stimulation in the neuron shown in H. Note the frequency-dependent depression of the EPSCs. Abbreviations: LM, stratum lacunosum moleculare; Or., stratum oriens; Pyr., stratum pyramidale; Rad., stratum radiatum.

Fig. S3

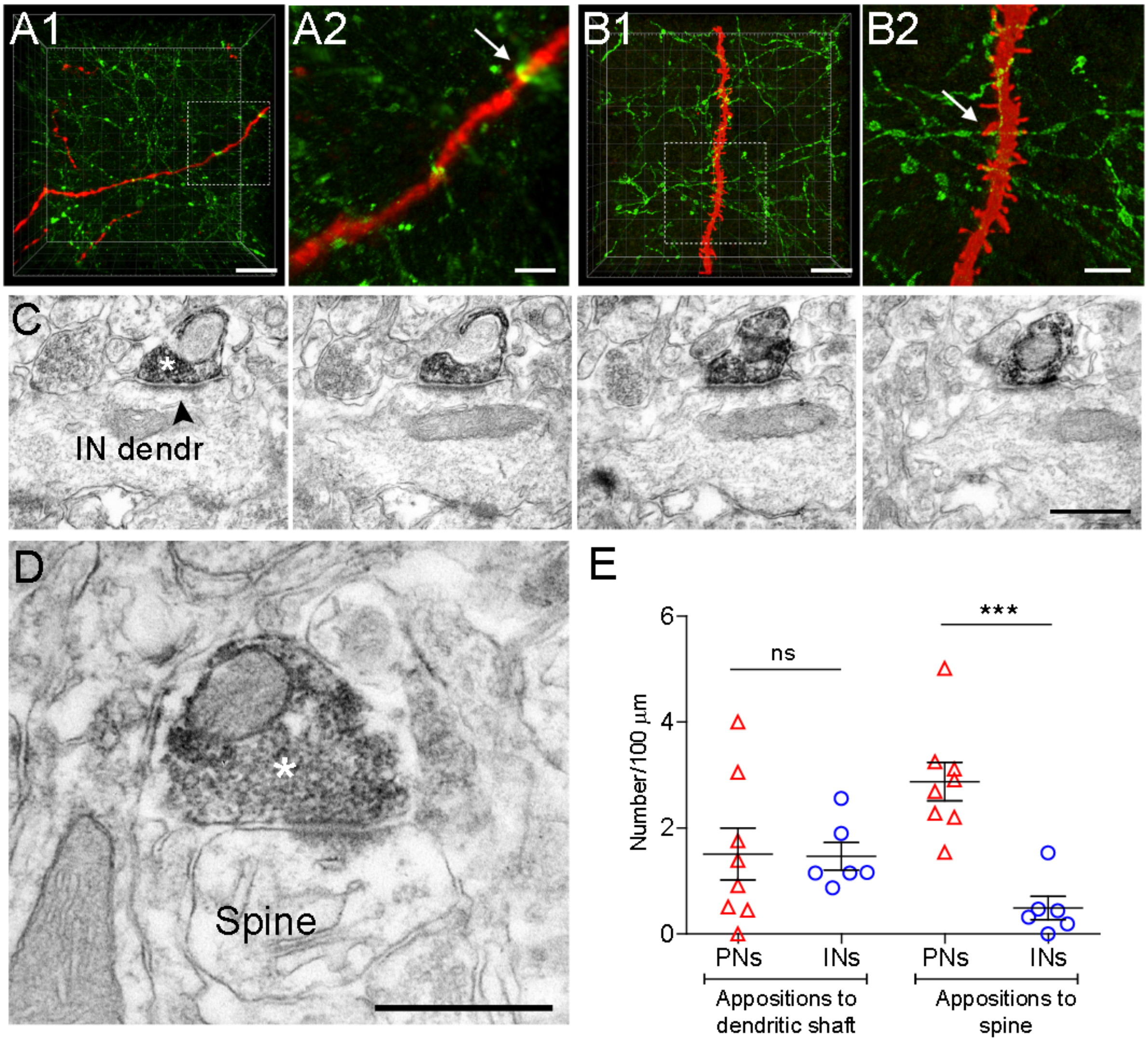

A1
A2
B1
B2
C
IN dendr
D
Spine
E
ns
***
Number/100 µm
6
4
2
0
PNs
INs
PNs
INs
Appositions to
dendritic shaft
Appositions to
spine

Figure S3, related to Fig. 2-8

CA1 pyramidal cells axons form asymmetric synapses onto both INs and PNs of the BA.

A, low and high magnification micrographs of a Huygens-deconvoluted confocal stack (z-stack: 24 μm; step size: 0.4 μm) of a representative aspiny dendrite of a recorded and biocytin-filled IN (shown in red). The fluorescence of the YFP (shown in green) reveals CA1 hippocampal axons and terminals. The arrow indicates an apposition to the dendritic shaft. **B**, low and high magnification micrographs of a Huygens-deconvoluted confocal stack (z-stack: 14 μm) of a representative spiny dendrite of a recorded and biocytin-filled PN (shown in red). The arrow indicates an apposition of a hippocampal bouton to a spine of the PN. **C**, serial electron micrographs of a green fluorescent protein (GFP)-immunolabeled hippocampal axon terminal forming a Type I (asymmetric) synaptic contact (arrowhead) with the dendritic shaft of a putative interneuron (IN dendr). Asterisk, note the diffuse electron-dense nickel-diaminobenzidine (NiDAB)-labeling that fills the entire bouton. **D**, GFP-immunolabeled hippocampal axon terminal (asterisk) forming a Type I (asymmetric) synaptic contact with a PN spine. **E**, density of appositions of CA1 hippocampal axons to IN ($n = 6$) and PN ($n = 8$) dendritic shafts and spines (per 100 μm). INs and PNs did not show any significant difference ($p > 0.5$) in the density of appositions to dendritic shafts. Conversely, PNs had a significantly higher density of appositions to spines compared to INs (***$p < 0.001$). Scale bars: A1 10 μm; A2 3 μm; B1 10 μm; B2, 3 μm; C-D, 500 nm. Data are presented as means ± SEM.

Fig. S4

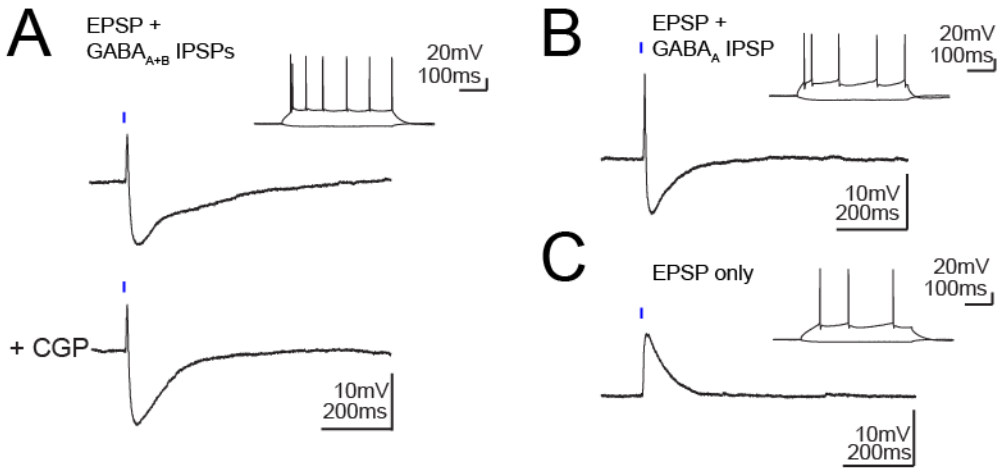

Fig.S4, related to Fig. 3, 5 and 7

Optogenetic stimulation of vCA1 axons evokes heterogeneous responses in BA PNs.

A, single light pulse stimulation of vCA1 axons triggered an excitatory postsynaptic potential (EPSP) followed by an early $GABA_A$ inhibitory postsynaptic potential (IPSP) and late $GABA_B$ IPSP in 66% (80/122) of PNs (*top*). The late $GABA_B$ IPSP was blocked by CGP54626 (5 μM, n = 26, *bottom*). **B**, an EPSP followed by an early IPSP was evoked in 15% (18/122) of the PNs. **C**, an EPSP alone was evoked in 16% of PNs (19/122). Furthermore, a few PNs (3/122) displayed only $GABA_A$ IPSP, one PN showed a $GABA_{A+B}$ IPSP, and another PN did not show any detectable response (not shown). Finally, hetereogeneous responses were also recorded in INs. Most of them (61%) displayed EPSPs only (27/44). We also observed: EPSP+$GABA_A$ IPSP (3/44); EPSP+$GABA_{A+B}$ IPSP (4/44); $GABA_A$ IPSP only (5/44); no response (5/44) (data not shown).

Fig. S5

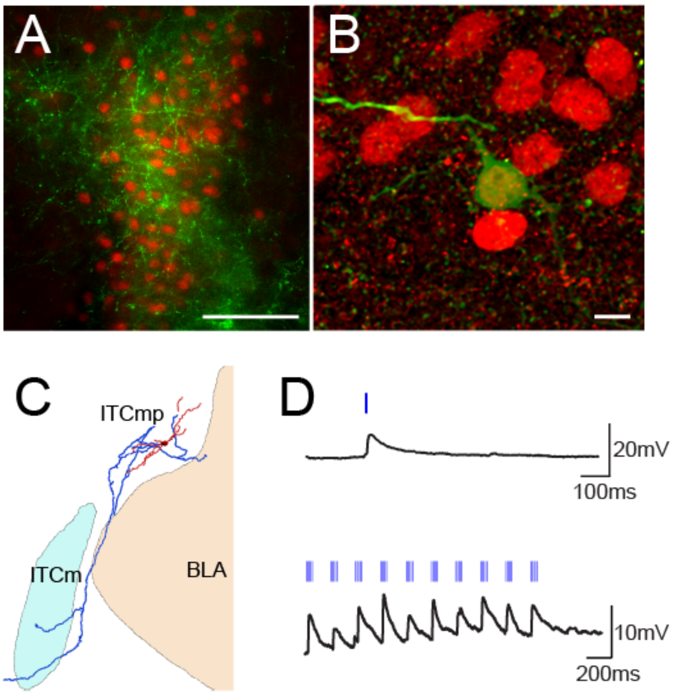

Fig. S5 ITC neurons respond to vCA1 stimulation, related to Fig. 3 and 5.

A, immunofluorescence micrograph of vCA1 hippocampal axons expressing ChR2-YFP (shown in green) coursing through the dorsomedial cluster of intercalated (ITCs) neurons identified for their nuclear expression of the transcription factor FoxP2 (shown in red). **B**, high magnification micrograph of a Huygens-deconvoluted confocal stack (z-stack: 26 μm; the z-step size was 0.46 μm) of the soma of a representative recorded and biocytin-filled medium spiny ITC neuron (shown in green) identified by its FoxP2 immunolabeling (shown in red). Fluorescence of the YFP tag of ChR2 in vCA1 hippocampal axons and terminals is visualized here also in the green channel. **C**, collapsed image of a 3D Neurolucida reconstruction of a recorded and biocytin-filled medium spiny ITC neuron revealing axonal branches running along the intermediate capsule. Soma and dendrites are shown in red, axon in blue. **D**, optogenetic activation of vCA1 axons evoked EPSPs in a representative ITC neuron. *Top,* single light pulse (3 ms). *Bottom*, Theta burst stimulation (3 ms light pulses). Scale bars: A, 50 μm; B, 5 μm. Abbreviations: BLA, basolateral amygdala; ITCmp, medial paracapsular ITC nucleus; ITCm, main ITC nucleus.

Fig. S6

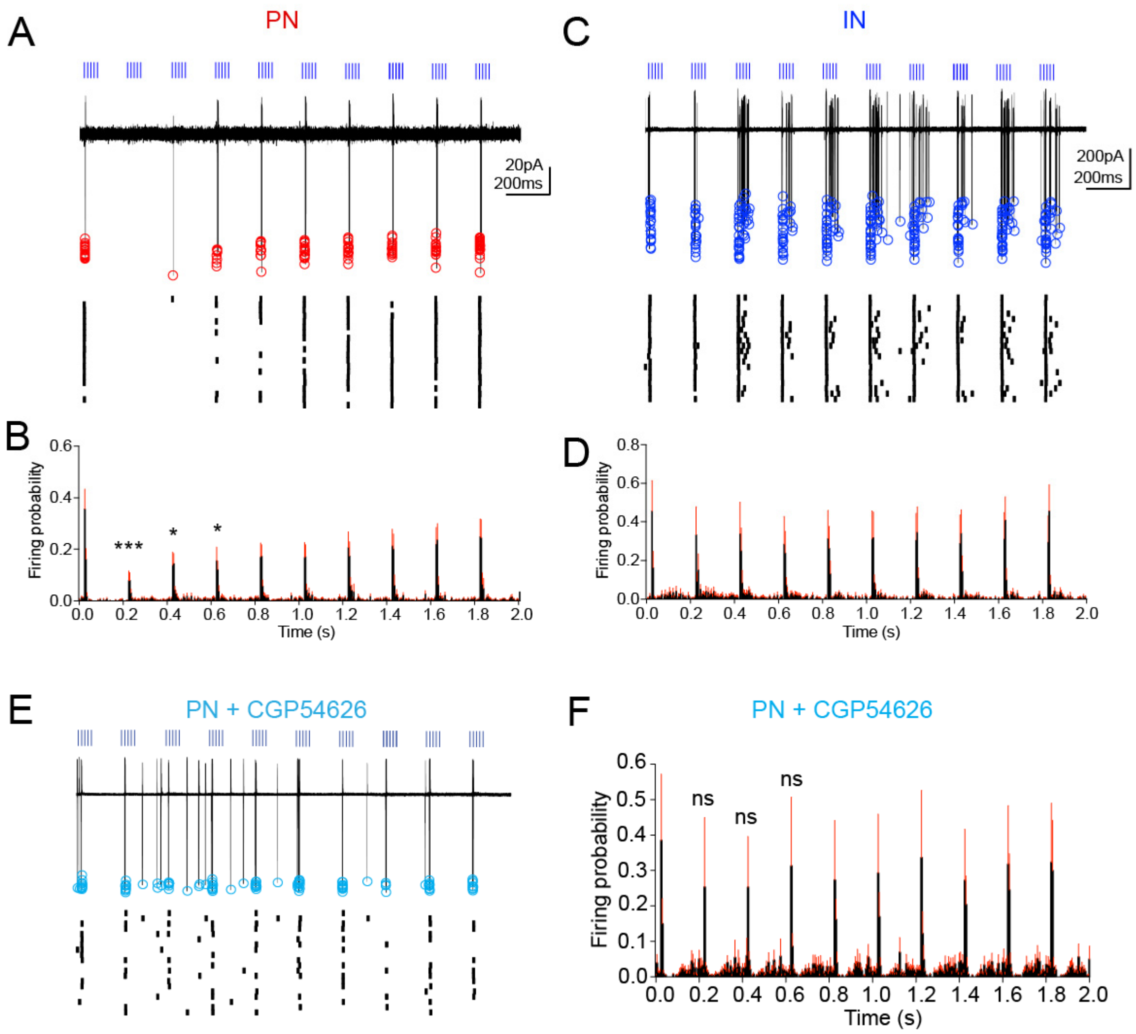

A
PN
20pA
200ms
B
Firing probability
0.0
0.2
0.4
0.6
***
*
*
0.0 0.2 0.4 0.6 0.8 1.0 1.2 1.4 1.6 1.8 2.0
Time (s)
C
IN
200pA
200ms
D
Firing probability
0.0
0.2
0.4
0.6
0.8
0.0 0.2 0.4 0.6 0.8 1.0 1.2 1.4 1.6 1.8 2.0
Time (s)
E
PN + CGP54626
F
PN + CGP54626
Firing probability
0.0
0.1
0.2
0.3
0.4
0.5
0.6
ns
ns
ns
0.0 0.2 0.4 0.6 0.8 1.0 1.2 1.4 1.6 1.8 2.0
Time (s)

Fig. S6, related to Fig. 4

High intensity TBS of vCA1 axons transiently depresses the firing of PNs in a $GABA_B$ receptor manner, and reliably activates INs *ex vivo*.

A, representative *ex vivo* cell attached recording from a PN during TBS (20 superimposed sweeps, *top*, singularly represented in rasterplot, *bottom*). The first train of light pulses (> 10 mW/mm^2) reliably evoked spikes, while the firing probability was reduced in the following 3 trains. Red circles denote spike negative peak. **B**, pooled data showing firing probability over time during TBS (bin = 5 ms, black: mean, red: SEM; n = 22, * $p < 0.05$, *** $p < 0.001$). **C**, representative cell attached recording from an IN during TBS (20 superimposed sweeps, *top*, singularly represented in rasterplot, *bottom*). TBS (> 10 mW/mm^2) reliably evoked spikes throughout all the 10 TBS trains. Blue circles denote spike negative peak. **D**, pooled data showing firing probability over time during TBS (bin = 5 ms, black: mean, red: SEM; n = 9, $p > 0.05$). **E,** representative cell attached recording from a PN during TBS applied at high intensity (> 10 mW/mm^2) in presence of CGP54626 (*top*, 20 superimposed sweeps; *bottom*, rasterplot). Blockade of $GABA_B$ receptors prevents the reduction in firing probability. **F**, mean firing probability over time during TBS in the presence of CGP54626 (bin = 5 ms, black: mean, red: SEM; $p > 0.05$, n = 10). Data are presented as means ± SEM.

Fig. S7

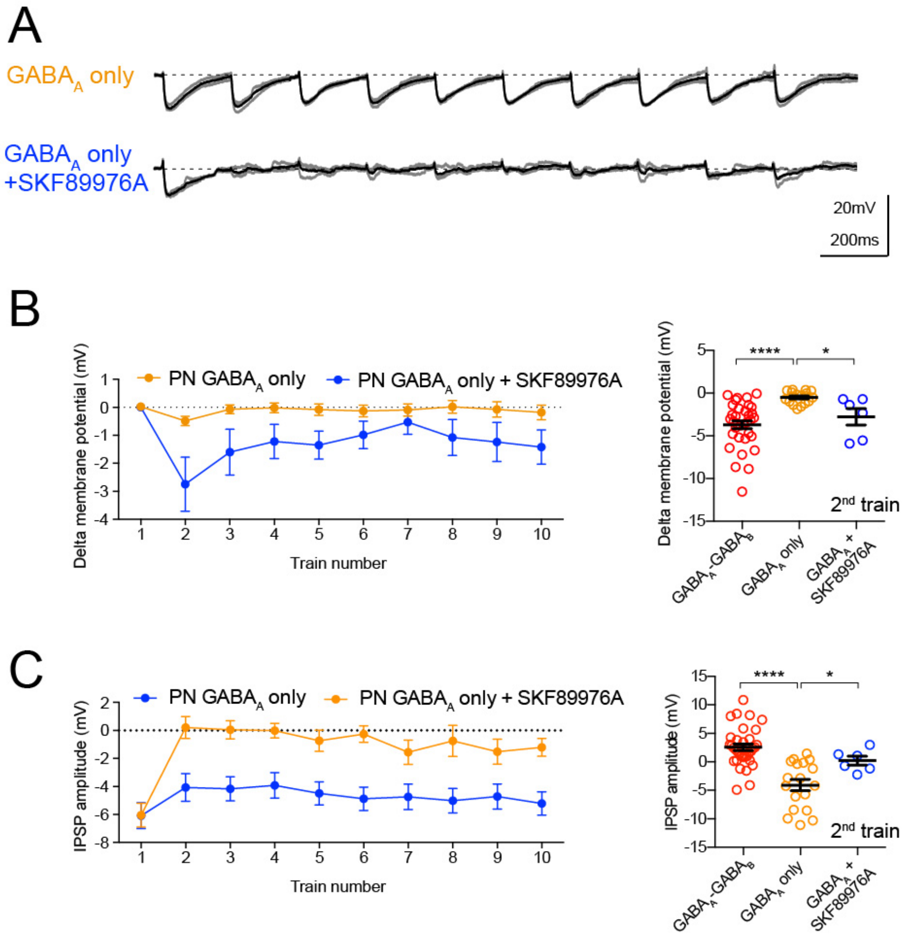
A
GABAA only
GABAA only
+SKF89976A
20mV
200ms
B
PN GABAA only
PN GABAA only + SKF89976A
Delta membrane potential (mV)
Train number
****
*
2nd train
GABAA-GABAB
GABAA only
GABAA + SKF89976A
C
PN GABAA only
PN GABAA only + SKF89976A
IPSP amplitude (mV)
Train number
****
*
2nd train
GABAA-GABAB
GABAA only
GABAA + SKF89976A

Fig. S7, related to Fig. 5 and 7

Blockade of GABA re-uptake evokes depression of FFI in PNs with vCA1-evoked $GABA_A$ receptor only IPSPs.

A, representative synaptic potentials (3 superimposed sweeps, grey traces; average, black trace) evoked by TBS in a PN were a $GABA_A$ only IPSP was induced by single light pulses (*top*). Same protocol delivered in presence of the GABA uptake blocker SKF89976A (25 μM, *bottom*). **B**, *left*, membrane potential recorded from PNs for each TBS train in control (n = 34) and in the presence of SKF89976A (n = 18). *Right*, membrane potential was significantly more depolarized during the 2nd train in PNs where TBS evoked $GABA_A$ and $GABA_B$ IPSPs than in PNs where a $GABA_A$ IPSPs only were evoked. SKF89976A significantly increased membrane hyperpolarization in PNs where a $GABA_A$ IPSPs only were evoked (n = 6). **C**, *left*, IPSP amplitude recorded from PNs for each TBS train in control (n = 9) and in the presence of SKF89976A (n = 6). *Right*, IPSP amplitude was significantly reduced during the 2nd train in PNs where TBS evoked $GABA_A$ and $GABA_B$ IPSPs, compared to PNs where a $GABA_A$ IPSPs only were evoked. However, TBS depressed $GABA_A$ IPSP only recorded in PNs in the presence of SKF89976A (n = 6). * $p < 0.05$; **** $p < 0.0001$. Data are presented as means ± SEM.

Fig. S8

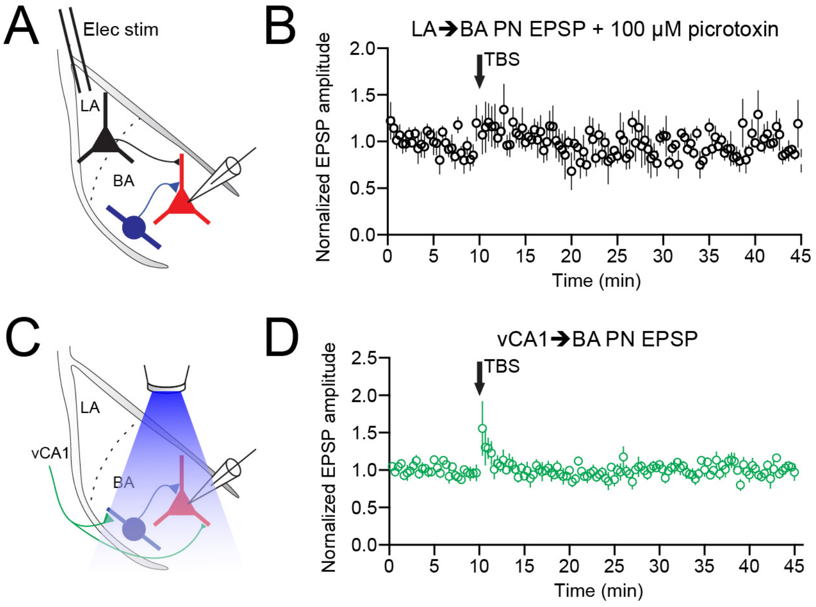

Fig. S8, related to Fig. 8

Blockade of $GABA_A$ receptors does not unmask LA-BA PN LTP, and TBS of vCA1 axons does not evoke vCA1-BA PN LTP.

A, schematic of the experimental configuration: the lateral amygdala (LA) was stimulated electrically while recording from BA PNs. **B**, LA-BA PN EPSP amplitude plotted over time (stimulation occurs every 10 s). Electrical TBS of the LA did not evoke long term potentiation (LTP) at LA-BA PN synapses in presence of the $GABA_A$ receptor antagonist picrotoxin (100 µM, n = 5). **C**, schematic of the experimental configuration: vCA1 axons were stimulated optically while recording from BA PNs. **D**, vCA1-BA PN EPSP amplitude plotted over time (stimulation occurs every 10 s). Optical TBS of vCA1 axons evoked a short term facilitation of the vCA1-BA PN EPSP, but no LTP (n = 5). Data are presented as means ± SEM.

Supplemental Experimental Procedures

***In vivo* recordings, photostimulation and analysis**

C57BL/6J or CaMKIIα-Cre mice+/+ (2-4 months old) were anesthetized with intraperitoneal injections of urethane (1.5 g/kg body weight). For the duration of the surgery (~2 hours), urethane anesthesia was supplemented with 0.5% isoflurane in oxygen (2 L/min). Isoflurane was switched off at least 1 hour before recordings. Supplemental injections of 10% the initial dose of urethane were administered when needed (approximatively every 2 hours). Body temperature was measured with a rectal probe, and maintained at 37°C using a homeothermic heating device (Harvard apparatus). Craniotomies were drilled above the right hippocampus (HPC) and amygdala. To monitor the firing of amygdala neurons in relation to hippocampal slow wave activity (SWA) and theta oscillations, extracellular recordings were performed from the BA and the dorsal CA1 using separate glass pipettes filled with 3% Neurobiotin (Vector Laboratories) in 0.5 M NaCl (10–18 MΩ resistance *in situ*). Glass electrode signals were referenced against a wire implanted subcutaneously in the neck. To monitor oscillatory activity in the TeA, an electrocorticogram (ECoG) was recorded using a 1 mm diameter steel screw implanted at -4.2 mm posterior, +4.3 mm lateral from bregma. Glass electrode signals were amplified (ELC-01MX, NPI Electronic Instruments) and differentially filtered (DPA-2FS, NPI Electronic Instruments) to extract local field potentials (LFPs, 200x amplification, 0.3-5000 Hz filtering) and unit activities (10x amplification, 300-5000 Hz filtering). Raw ECoG signal was bandpass filtered (0.3–1500 Hz) and amplified (2000x). All signals were digitized online at 20 kHz using a Power 1401 analog-digital converter (Cambridge Electronic Design) and stored on a PC running Spike2 software (Cambridge Electronic Design). To photostimulate vCA1 pyramidal cells axons innervating the BA, a fiber optic cannula (200 µm core diameter, 0.39 NA, Thorlabs) connected to a Luxx 488-200 diode laser (Omicron) was implanted 0.5-0.7 mm above the recording site (inserted -0.5 mm posterior, +3 mm lateral from bregma, and advanced 4 mm at a 16° angle ventral from the brain surface, Fig. S1D). Light power at the fiber optic tip was measured using a PM100D power meter and photodiode

sensor (Thor Labs). TBS protocol consisted of 50 trains of 5 light pulses (3 ms duration, 19 ± 1.2 mW at fiber optic tip) delivered at 10 ms intervals (100 Hz), with 200 ms between trains. TBS was delivered every 20 s and was repeated for 8-15 trials for each neuron. After recordings, 14/27 PNs and 10/25 INs were filled with Neurobiotin using juxtacellular labeling (Bienvenu et al., 2012; Bienvenu et al., 2015; Pinault, 1996). The position of neurons that could not be labeled after recording was calculated using a labeled reference neuron in the same brain. In order to verify the location of the hippocampal electrode, an extracellular Neurobiotin deposit was made in the dorsal CA1 (100 nA anodal current 1 s, 50% duty cycle for 20-30 min).

All data were analyzed off-line using Spike2 built-in functions and custom scripts (Tukker et al., 2007) and MATLAB (Mathworks, Inc.) custom scripts. Spike2 clustering function supervised manually was used to isolate single units, and identity of labeled neurons was systematically ensured as described above. Before immunohistochemically determining whether labeled neurons were PNs or INs, putative PNs were separated from putative INs using *K-means* supervised clustering for 3 parameters: first positive peak of the spike to negative peak (P1-P2), negative peak to second positive peak (P2-P3) and the mean CV2, a measure of firing regularity (Fig. S1A-C). Theta epochs, occurring during activated cortical state, were isolated when the ratio of theta (3–6 Hz) to delta (2–3 Hz) power in 10 s windows of the dCA1 or TeA LFP was greater than 4 (Csicsvari et al., 1999; Klausberger et al., 2003). SWA epochs were defined if the theta/delta ratio was lower than 2. The mean firing rate of a given neuron was calculated by summing all spikes during theta or SWA epochs and dividing this by the sum of durations of the epochs. Only neurons that had stable spontaneous firing rates/patterns and stable spike widths, and did not display any 'injury discharge' were included. To analyze responses to optogenetic TBS, peri-stimulus time histograms of firing rates (PSTHs, 50 ms bins) were generated for each trial (8-15 trials in total). PSTHs were normalized by dividing binned frequencies to the pre-TBS baseline mean (5 s). A neuron was considered optically modulated if the mean firing rate in the 50 ms bins during TBS light trains differed statistically from the mean baseline. To obtain population PSTHs,

normalized PSTHs were generated from all trials from each neuron and averaged. The following rules were defined, based on histological verifications: 1) recorded neurons had to be located in the BA nucleus (either by observation of Neurobiotin filled neurons or by calculating the position of the recorded unlabeled neurons according to the position of a reference labeled neuron); 2) transfection with ChR2 had to be restricted to ventral/intermediate CA1; 3) the BA had to be innervated by a pattern of ChR2+ axons as shown in Fig. 2B; 4) the fiber optic tip had to be located above the BA and ~500 μm above the recording site. If these criteria were not met, neurons were discarded and not analyzed.

***Ex vivo* recordings, photostimulation and analysis**

After allowing 3-4 weeks for ChR2 expression, mice (postnatal day 40-60) were decapitated under deep isoflurane anesthesia (4% in O_2), and their brains were rapidly removed and placed in ice-cold sucrose-containing artificial cerebrospinal fluid (ACSF) cutting solution containing (in mM): 75 sucrose, 87 NaCl, 25 $NaHCO_3$, 2.5 KCl, 1.25 NaH_2PO_4, 0.5 $CaCl_2$, 7 $MgCl_2$, 25 glucose, saturated with 95% O_2, 5% CO_2, at pH 7.3-7.4. Slices (325 μm thickness) including amygdala were cut (Microm HM 650 V, Thermo Fisher Scientific Inc., Germany) and transferred on a nylon mesh where they were maintained in a chamber initially containing sucrose ACSF cutting solution at 37°C for 30 min. During this period the cutting solution was gradually substituted (5 ml/min) with normal ACSF consisting of (in mM): 130 NaCl, 24 $NaHCO_3$, 3.5 KCl, 1.25 NaH_2PO_4, 2.5 $CaCl_2$, 1.5 $MgSO_4$, 10 glucose, saturated with 95% O_2, 5% CO_2, at pH 7.3. Slices were then stored at room temperature (18-22°C). Acute slices were secured under a nylon mesh, submerged and superfused with normal ACSF in a chamber mounted on the stage of an upright microscope (Axioskop, Zeiss, Jena, Germany). Slices were visualized with a 40×/0.1 NA water-immersion objective coupled with infrared and differential interference contrast (DIC) optics linked to a video camera (CCD Camera Orca R2, Hamamatsu, Hamamatsu City, Japan). Somatic whole-cell patch-clamp recordings (at ~33°C) were made from visually identified cells using borosilicate glass capillaries (GC120F, 1.2 mm,

100 o.d., Clarke Electromedical Instruments, Reading, UK), pulled on a DMZ puller (Zeitz-instrumente GmbH, Munich, Germany) and filled with a filtered intracellular solution consisting of (in mM): 140 K-gluconate, 4 KCl, 4 ATP-Mg, 0.3 GTP-Na_2, 10 Na_2-phosphocreatine, 10 HEPES and 0.5% w/v biocytin, osmolarity 270–280 mOsmol/L without biocytin, pH 7.3 adjusted with KOH. A cesium-based intracellular solution was used in some experiments to block K^+ channels consisting of (in mM): 42 Cs-methansulfonate, 88 CsCl, 10 HEPES, 10 Na_2-Phosphocreatine, 4 Mg-ATP, 0.3 Na-GTP and 0.5% w/v biocytin (all from Sigma-Aldrich), osmolarity 270–280 mOsmol/L without biocytin, pH 7.3 adjusted with CsOH. Resistance of the patch pipettes was 5-6 MΩ. Recordings were accepted only if the initial seal resistance was greater than 1 GΩ and series resistance did not change by more than 20% throughout the recording period. No correction was made for the junction potential between the pipette and the ACSF. For cell attached recordings, glass electrodes were filled with 150 nM NaCl. Suction of the membrane was applied to achieve a loose-patch configuration (≤ 90 MΩ seal). Recordings were performed in voltage clamp mode by setting the pipette potential to obtain 0 pA of membrane current (Alcami et al., 2012). To induce firing in PNs, we applied a positive pipette current (10-1000 pA). For unlabeled neurons recorded in whole cell mode, PNs were distinguished from INs according to their lower input resistance, higher membrane constant, smaller fast afterhyperpolarization and adapting, <20 Hz maximum firing rates (Sosulina et al., 2006). All electrophysiological signals were amplified (10 mV pA^{-1}, EPC9/2 amplifier HEKA Electronik, Lambrecht, Germany, PULSE software), low pass filtered at 2.9 kHz, digitized at 5 or 10 kHz. The amplifier was controlled with the PULSE data acquisition and analysis program (HEKA).

Optical stimulation of hippocampal ChR2-expressing afferents in the BA was performed using an optoLED system (Cairn Research), consisting of a 470 nm, 3.5 W LED mounted on a Zeiss Axioskop 2 FS microscope, to give 0.1 or 3 ms duration light pulses of ~5% of maximum output power. The steady-state light power at the tissue was measured using a PM100D power meter and photodiode sensor (Thor Labs). The spot size corresponded to the area of the slice visualized using a 40x/0.8 NA water

immersion objective, i.e. approximately 200 μm. Optical TBS consisted of 10 trains of 5 light pulses (3 ms duration) delivered at 10 ms intervals (100 Hz), with 200 ms between trains. TBS was delivered every 20 s and was repeated at least 10 times.

Inhibition of firing after single light stimulation observed in cell attached mode experiments was analyzed by comparing the mean firing probability occurring between 100 and 200 ms after the stimulation. The PSPs were defined as such if their amplitude was 2 standard deviations above or below the mean baseline. The EPSP peak was calculated throughout the 10 TBS trains using the pre-1st train membrane potential as baseline. The PSP amplitude was calculated from the pre-train membrane potential (baseline) to the peak of the PSP evoked by light pulses. The PSPs were not analyzed in neurons in which vCA1 stimulation evoked spikes to accurately determine their peak amplitude. The membrane potential for each train of the TBS was measured as the mean membrane potential across 20 ms preceding each train. To determine the E_{IPSC}, I/V curves were constructed measuring the IPSC peak amplitude during the 1st and 2nd TBS trains for -90 → +50 mV (5 mV steps) holding potentials. Linear regression was used to calculate a best-fit line for this relationship for each cell. To calculate E_{IPSC} and slope conductance, the interpolated intercept of the linear fit with the abscissa and the slope of the linear fit were taken, respectively. Electrical stimulation was performed with a bipolar tungsten electrode. Evoked PSPs were recorded in whole-cell current-clamp mode at -65 mV. Evoked IPSCs were recorded in voltage clamp mode at -50 mV. Electrical TBS consisted of trains of five stimuli (0.5 ms) at 10 ms intervals (100 Hz), with 200 ms between trains. The pairing-induced plasticity protocol consisted of pairing 50 optical TBS (ChR2+ vCA1 axons) trains with 50 electrical TBS (LA stimulation). The LA stimulation was set with delays of 10 ms or 100 ms from the optogenetic stimulation in order to evoke an EPSP from the LA input at the peak of the IPSP triggered by the vCA1 input, or when the IPSPs were virtually extinguished, respectively.

Histological analyses

Processing of sections recorded ex vivo

Following intracellular recording *ex vivo*, slices were fixed overnight in 4% paraformaldehyde and 15% saturated picric acid in 0.1 M phosphate buffer (PB; pH 7.4) at 4ºC. Sections were embedded in a block of 10% gelatine (Merck, Darmstadt, Germany) in deionized water, postfixed in 4% paraformaldehyde (PFA; Agar Scientific Ltd., Stansted, UK) in 0.1M PB including 1.25% glutaraldehyde (Polysciences Inc., Warrington, PA, USA) for 30 min, washed two times and then re-cut in 60 µm thick consecutive sections on a vibratome (Leica VT 1000 S; Leica Microsystems, Vienna, Austria). The sections were collected in consecutive order in 0.1 M PB and stored at 6°C until further usage. Biocytin-labeled neurons were confirmed as PNs or INs according to their dendritic and axonal patterns: PNs displayed larger, pyramidal-shaped somata, thick spiny dendrites and projected outside the BLA. INs had usually smaller, ovoid somata, spine sparse dendrites and their axon branched profusely within the BLA.

Camera lucida reconstructions were performed using a drawing tube mounted onto a Zeiss Axioplan2 microscope with a 63x oil 1.4 NA objective. Amygdala nuclei boundaries were drawn from one of the 2 sections from which the axon was reconstructed. We used adjacent coronal sections and, in several instances immunohistochemistry, to determine boundaries between LA and BA nuclei.

Processing of whole brains

Mice were transcardially perfused with saline followed by 4% paraformaldehyde w/v, 15% saturated picric acid v/v, in 0.1 M phosphate buffer. If the brain contained juxtacellularly labeled neuron(s), perfusion took place one to four hours after cell labeling. Brains were sectioned using a vibratome (Leica VT 1000 S) into 60 µm thick slices. Sections were stored 0.1M PB containing 0.05% sodium azide until further usage.

Immunohistochemistry

The expression of YFP in hippocampal fibres was visualized by incubating sections in 1:1000 chicken anti-GFP (Aves Labs) in TBS containing 0.1% Triton X-100 and 1% NDS overnight at 4ºC followed by 1:500 donkey-anti-chicken-488 fluorophore (Jackson

Immunoresearch) in TBS containing 0.1% Triton X-100 for 2 hours at room temperature. To facilitate anatomical characterization, the sections were incubated for 30 min in a 1:200 Nissl-Cy5 stain (Neurotrace, Invitrogen). For visualization of neurons labeled *ex vivo*, free-floating sections were washed in TBS and then blocked in 20% NGS in TBS containing 0.1% Triton X-100 for 2 hours at room temperature. After blocking, the sections were directly incubated in streptavidin-cyanine 3 (Cy3) (1:1000; Vector Laboratories), to visualize the biocytin, for 72 hours at 6°C diluted in a solution made of TBS containing 2% NGS and 0.1% Triton X-100. For visualization of neurons labeled *in vivo,* sections were incubated in streptavidin-Cy3 (1:1000-3000) diluted in a solution of PBS containing 0.3% Triton X-100 for 3 hours at room temperature. After washing with TBS/PBS the sections were mounted on glass slides and coverslipped with Vectashield (Vector Laboratories) as embedding medium. The exposure to light was kept at a minimum level for the whole procedure. To assess immunoreactivity of the neurons labeled *in vivo*, sections were blocked in 10% NDS in PBS containing 0.3% Triton X-100 and then incubated with goat anti-CaMKIIα (1:200, Santa Cruz, #sc-5391) or guinea pig anti-VGAT (1:500, #131004) antibodies. Following washes in PBS, sections were incubated in Alexa 647-conjugated secondary antibodies (1:250, Jackson Immunoresearch).

Electron microscopy

Pre-embedding immunocytochemistry experiments were carried out according to previously published procedures with minor modifications (Sreepathi and Ferraguti, 2012). Briefly, cryoprotected free floating sections were freeze-thawed twice to allow antibody penetration. After blocking unspecific binding sites with 20% NGS in TBS for 2 hours at room temperature, sections were incubated for ~72 hours at 6°C with a rabbit anti-GFP antibody (diluted 1:1000; Molecular Probes, cat. no. A-11122) made up in a solution containing 2% NGS in TBS, and then overnight at 6°C with an anti-rabbit biotinylated secondary antibody (diluted 1:100; Vector). Antigen-antibody complexes were visualized through horseradish peroxidase (HRP)-based reaction. Sections were

contrast-enhanced with 2% OsO_4 in 0.1M PB for 40 min at RT and 1% uranyl-acetate in 50% ethanol for 30 min at RT, making sure they were protected from light. After dehydration with graded ethanol and propylene oxide, sections were embedded in epoxy resin (Durcupan ACM-Fluka, Sigma, Gillingham, UK). Serial ultrathin sections (70 nm) were cut using a diamond knife (Diatome, Biel, Switzerland) on an ultramicrotome (EM UC7, Leica, Vienna, Austria), collected on copper slot grids coated with pioloform (Agar, Stansted, England) and analyzed with a transmission electron microscope (Philips CM120) equipped with a Morada CCD camera (Soft Imaging Systems, Münster, Germany). Images were level adjusted and cropped in Photoshop (Adobe) without changing any specific feature within.

SDS-FRL was performed according to previously published procedures with minor modifications (Kasugai et al., 2010). The brains of adult mice (C57BL/6 mice) were perfusion-fixed with PB (0.1 M, pH 7.4) containing 1% formaldehyde, and 15% of a saturated solution of picric acid. Blocks containing the amygdala were cut into 140 μm coronal sections by a Leica VT1000S Vibratome and the BA was dissected out under a stereomicroscope. BA tissue blocks were cryoprotected with 30% glycerol in 0.1 M PB overnight at 4°C, high-pressure frozen (HPM 010; Bal-Tec, Balzers, Liechtenstein), fractured and replicated in a freeze-etching device (BAF 060; Bal-Tec). Fractured faces were replicated by rotary deposition of carbon (5 nm) evaporated with an electron beam gun positioned at a 90° angle, shadowed unidirectionally by platinum-carbon (2 nm) with the gun positioned at a 60° angle, followed by an additional carbon layer (15 nm) applied from a 90° angle. Tissue was solubilised in a solution containing 2.5% SDS and 20% sucrose made up in 15 mM Tris buffer, pH 8.3, at 80°C on a shaking platform for 18 hours. Replicas were kept in the same solution at room temperature until processed further. On the day of immunolabeling, replicas were washed in 25 mM TBS containing 0.05% BSA and incubated in a blocking solution containing 5% BSA in 25 mM TBS for 1h. Subsequently, the replicas were sequentially incubated in primary antibodies (affinity purified polyclonal rabbit antiserum against $GABA_B$ diluted 1:200, kindly donated by Prof. R. Shigemoto, followed by a polyclonal Guinea pig antiserum against the rat vesicular

GABA transporter purchased from Synaptic Systems, cat no. 131 044, diluted 1:300). After several washes, the replicas were sequentially reacted overnight at room temperature with gold-coupled secondary antibodies (goat anti-rabbit 5 nm followed by goat anti Guinea pig 10 nm, purchased from British Biocell International both diluted at 1:30) made up in 25 mM TBS and containing 5% BSA. Replicas were then washed, picked up on 100-line copper grids and analyzed using a transmission electron microscopy imaging system (CM120 TEM, Morada CCD camera). To test for cross reactivity of gold-conjugated secondary antibodies in co-localization studies, control replicas were reacted with one of the primary antibodies and a mixture of secondary antibodies conjugated to different sizes of gold and directed against different species. No non-specific cross reactivity of gold-conjugated secondary antibodies was observed.

Confocal microscopy

The sections were analyzed with a confocal microscope (TCS SP5 confocal, Leica Microsystems, Vienna, Austria) using the LAS-AF software (Leica) for acquisition. To excite the fluorophores ChR2-YFP and Cy3, we used the 488 nm line of an argon laser and the 561 nm line of the DPSS laser, respectively. Images of Cy3 positive dendrites were collected from biocytin injected neurons. Confocal images were deconvoluted with the Huygens software (Scientific Volume Imaging, Hilversum, Netherlands), and analyzed with Imaris 7.1 (Bitplane, Zürich, Switzerland). In 3D images, ChR2-YFP positive boutons were observed to form appositions with dendrites of filled neurons. The identification of putative synapses was based on the finding that YFP labeled boutons were always in close proximity to a labeled dendrite/spine when viewed from different perspectives. The length of the observed dendrites was also measured with Imaris.

Supplemental References

Alcami, P., Franconville, R., Llano, I., and Marty, A. (2012). Measuring the firing rate of high-resistance neurons with cell-attached recording. J Neurosci *32*, 3118-3130.

Bienvenu, T.C., Busti, D., Magill, P.J., Ferraguti, F., and Capogna, M. (2012). Cell-type-specific recruitment of amygdala interneurons to hippocampal theta rhythm and noxious stimuli in vivo. Neuron 74, 1059-1074.

Bienvenu, T.C., Busti, D., Micklem, B.R., Mansouri, M., Magill, P.J., Ferraguti, F., and Capogna, M. (2015). Large intercalated neurons of amygdala relay noxious sensory information. J Neurosci 35, 2044-2057.

Csicsvari, J., Hirase, H., Czurko, A., Mamiya, A., and Buzsaki, G. (1999). Oscillatory coupling of hippocampal pyramidal cells and interneurons in the behaving Rat. J Neurosci *19*, 274-287.

Kasugai, Y., Swinny, J.D., Roberts, J.D., Dalezios, Y., Fukazawa, Y., Sieghart, W., Shigemoto, R., and Somogyi, P. (2010). Quantitative localisation of synaptic and extrasynaptic GABAA receptor subunits on hippocampal pyramidal cells by freeze-fracture replica immunolabelling. Eur J Neurosci *32*, 1868-1888.

Klausberger, T., Magill, P.J., Marton, L.F., Roberts, J.D., Cobden, P.M., Buzsaki, G., and Somogyi, P. (2003). Brain-state- and cell-type-specific firing of hippocampal interneurons in vivo. Nature *421*, 844-848.

Pinault, D. (1996). A novel single-cell staining procedure performed in vivo under electrophysiological control: morpho-functional features of juxtacellularly labeled thalamic cells and other central neurons with biocytin or Neurobiotin. J Neurosci Methods *65*, 113-136.

Sosulina, L., Meis, S., Seifert, G., Steinhauser, C., and Pape, H.C. (2006). Classification of projection neurons and interneurons in the rat lateral amygdala based upon cluster analysis. Mol Cell Neurosci 33, 57-67.

Sreepathi, H.K., and Ferraguti, F. (2012). Subpopulations of neurokinin 1 receptor-expressing neurons in the rat lateral amygdala display a differential pattern of innervation from distinct glutamatergic afferents. Neuroscience *203*, 59-77.

Tukker, J.J., Fuentealba, P., Hartwich, K., Somogyi, P., and Klausberger, T. (2007). Cell type-specific tuning of hippocampal interneuron firing during gamma oscillations in vivo. J Neurosci *27*, 8184-8189.
